# Overlooked role of heterotrophic prokaryotes in sulfur oxidation makes the sediment of the Bohai Sea a sufficient sink of hydrogen sulfide

**DOI:** 10.1128/mbio.01722-25

**Published:** 2025-07-07

**Authors:** Zhiyi Chen, Luying Xun, Yongzhen Xia, Xianzhe Gong

**Affiliations:** 1 State Key Laboratory of Microbial Technology, Shandong University, Qingdao, Shandong, China; 2 Institute of Marine Science and Technology, Shandong University, Qingdao, Shandong, China; 3 School of Molecular Biosciences, Washington State University 744660, Pullman, Washington, USA; 4 Southern Marine Science and Engineering Guangdong Laboratory, Guangzhou, Guangdong, China; 5 Department of Marine Science, Marine Science Institute, University of Texas at Austin, Austin, Texas, USA; University of Tennessee at Knoxville, Knoxville, Tennessee, USA

**Keywords:** sulfur oxidation, marine sediment, microbial diversity, metagenomics, metatranscriptomics

## Abstract

Marine sediments are the active sites for the biogeochemical cycling of sulfur. Sulfate is used as a major terminal electron acceptor for the anaerobic oxidation of organic compounds in deep sediments, and the produced sulfide is normally oxidized back to sulfate in upper sediments. However, it is unclear which microorganisms, metabolic pathways, and enzymes are mainly involved in oxidation. Here, we used metagenomics, metatranscriptomics, and the testing of sulfide, thiosulfate, and sulfite oxidation in sediment samples to figure out how sulfide is oxidized in the Bohai Sea sediments. Surprisingly, sulfur oxidation is widespread in the microbial community (>67.1%) of mostly heterotrophic prokaryotes across 44 phyla, dominated by Proteobacteria. Known chemolithotrophic sulfur oxidizers were absent. The prevalent sulfur-oxidizing pathway was sulfide to zerovalent sulfur, sulfite, and then sulfate. Thiosulfate is not a major metabolic intermediate. Genes encoding sulfide oxidation (*sqr* and *fccAB*), zerovalent sulfur oxidation (*pdo*, *rdsrAB*, and *shdr*), and sulfite oxidation (*aprAB*/*sat*) were abundant and upregulated after adding NaHS. Thiosulfate, which is formed between the reaction of zerovalent sulfur and sulfite, was only slowly oxidized, which was consistent with the lack of key genes encoding for direct oxidation of thiosulfate to sulfate. The findings indicate how sulfur is oxidized in the Bohai Sea sediments. The common participation in sulfur oxidation by most heterotrophic prokaryotes results in the effective oxidation of sulfide in the surface sediment, blocking the release of hydrogen sulfide into the water column.

**IMPORTANCE:**

Sulfur cycling is tightly interwoven with other crucial element cycles, including carbon, nitrogen, and iron in marine sediments. Sulfate is the most abundant electron acceptor in marine sediments, and sulfate reduction generates a large amount of sulfide. The majority of sulfide is oxidized to sulfate via abiotic or biological transformations, mainly by sulfur oxidizers with different redox states. However, autotrophic sulfur oxidizers, considered key players for sulfur oxidation, are in low abundance in the sediment, limiting our understanding of the pivotal biogeochemical process. This study shows the prevalent distribution of sulfur oxidation among the microbial community and emphasizes the importance of heterotrophic sulfur oxidation in sediments. It evidences the importance of previously overlooked key enzymes for elemental sulfur oxidation and supports that thiosulfate is not the major intermediate during sulfur oxidation. Understanding these key processes is crucial for elucidating biogeochemical processes in marine sediments.

## INTRODUCTION

The biogeochemical sulfur cycle is an essential process consisting of abiotically and biologically mediated reactions in marine sediments. The sulfur cycles interlink with other biogeochemical cycles, such as carbon and nitrogen cycles. On a global scale, sulfate reduction facilitates the oxidation of organic matter from 22 up to 77 Tmol of carbon per year in marine sediments (1, 2), which is associated with the production of a large amount of reduced sulfide. However, over 80% sulfide is eventually reoxidized back to sulfate, while less than 20% is ultimately buried as metal sulfides, e.g., FeS, FeS_2_, or sulfurized organic matter (3, 4). Sulfide oxidation from the most reduced state (−2, sulfide) to the most oxidized state (+6, sulfate) is primarily conducted by microorganisms, such as sulfur-oxidizing bacteria, with a range of intermediates, including zerovalent sulfur, thiosulfate, tetrathionate, and sulfite (5, 6). The common form of zerovalent sulfur is octasulfur (S_8_), which reacts with sulfide and organic thiols to generate inorganic polysulfide (HS_*n*_^−^, *n* ≥ 2) and organic polysulfide (RS_*n*_^−^, *n* ≥ 2) (7). Since S_8_, HS_*n*_^−^ and RS_*n*_^−^ are interchangeable, we collectively refer to them as zerovalent sulfur in this report.

Various microorganisms, including autotrophic and heterotrophic bacteria, oxidize sulfur (8). However, sulfur oxidation is still considered a specialized trait in the community. Heterotrophic bacteria, especially the Roseobacter clade, dominate sulfide oxidation in coastal waters and sediments (9). A wide phylogenetic spectrum of Flavobacteria and Proteobacteria oxidizes thiosulfate in marine sediments and hydrothermal vents (10). Since individual microbes possessing the gene sets for complete oxidation of sulfur from sulfide to sulfate are rare (11), sulfur oxidation relies on metabolic handoffs across different community members. This likely suggests that sulfur oxidation is dependent on metabolic interactions, stressing the importance of revealing the process of microbial-driven sulfur oxidation based on a holistic perspective of the entire community.

Several sulfur oxidation pathways associated with different key genes using various inorganic sulfur compounds as substrates have been identified. Sulfide is oxidized to zerovalent sulfur by membrane-bound sulfide:quinone oxidoreductase (SQR) in the cytoplasm (12) and flavocytochrome *c*:sulfide dehydrogenase (FCSD) in the periplasm (13). Flavocytochrome *c*:sulfide dehydrogenase consists of two proteins: FccB is the catalytic unit, and FccA is the cytochrome *c*. Zerovalent sulfur produced by FCSD in the periplasm is primarily converted to polysulfides using glutathione as the carrier. These polysulfides are transported into the cytoplasm via polysulfide transporters, such as polysulfides transmembrane transporters (PmpAB), rhodanese (Rhd), and tRNA 2-thiouridine synthesizing protein A (TusA) for further oxidation (14). Additionally, the intracellular S_8_ produced by SQR is directly transferred to other bacteria for metabolism because of its high solubility in the cell membranes and cell envelope polysaccharides (15). Zerovalent sulfur is oxidized to sulfite in the cytoplasm by reverse dissimilatory sulfite reductase (rDsrAB) (16), persulfide dioxygenase (PDO) (17), and sulfur-oxidizing heterodisulfide reductase (sHdr) (13, 18, 19). Specific sulfur transferases (TusA and DsrEFH) transfer sulfur to DsrC, forming protein-bound sulfur (DsrC-trisulfide), which is oxidized by rDsrAB (20). Furthermore, zerovalent sulfur can be disproportionated to sulfide, thiosulfate, and sulfite by cytoplasmic sulfur oxygenase reductase (SOR) (21, 22). PDO and SOR catalyze reactions that require molecular oxygen and do not allow energy conservation, while this is not the case for rDsr and sHdr (14). Sulfite is oxidized to sulfate by periplasmic sulfite oxidase (SUOX), also termed a “sulfite dehydrogenase” and present in all three domains of life (23–25), as well as by the periplasmic heterodimeric sulfite dehydrogenase (SorAB) (26), membrane-bound sulfite dehydrogenase (SoeABC) (27), adenylylsulfate reductase (AprAB), and sulfate adenylyltransferase (SAT) (13, 28). Alternatively, sulfite reacts with zerovalent sulfur to generate thiosulfate (29). Thiosulfate is oxidized by the periplasmic sulfur oxidation system (SoxABCDXYZ) (30) to sulfate or by periplasmic thiosulfate dehydrogenase (TsdA) and membrane-bound thiosulfate:quinone dehydrogenase (DoxD) to tetrathionate (13, 31, 32). Tetrathionate is converted by thiosulfohydrolase (SoxB) to sulfate and zerovalent sulfur (33) or by periplasmic tetrathionate hydrolase (TetH) to sulfate, thiosulfate, and zerovalent sulfur (13, 34). Thiosulfate and zerovalent sulfur are further metabolized by the enzymes mentioned above.

Despite a major process of the oxidation and reduction of sulfur in marine sediments, the associated dynamics, microbial diversity, and relevant biogeochemical impacts remain relatively underexplored (35–37). It is unclear which microorganisms are responsible for sulfur oxidation and which metabolic pathway is mainly used for sulfur oxidation. These insights are required to inform future predictions of microbial responses to the increased anthropogenic activities and changing oceans in coastal marine environments. The coastal sea has a high concentration of nutrients and organic matter as the energy sources, making coastal sediments “hotspots” for microbial activity and geochemical transformations (38). The Bohai Sea is a gulf/inland sea on the east coast of China with an average depth of only 18 meters (39). The high productivity in the overlaying water due to the rich nutrients and the shallow water column contribute to a large amount of buried organic matter in the sediment with a high sedimentation rate (40). Here, we selected 15 samples representing three layers of sediments from three sampling stations (M3, M8, and BHB10) in the Bohai Sea to untangle the sulfur-oxidizing communities with metagenomic sequencing, measurements of inorganic sulfur species, and test the oxidation rates of these species. Our findings revealed major participants and pathways of sulfur oxidation in the Bohai Sea sediments and advanced our understanding of sulfur biogeochemical cycling in marine sediments.

## RESULTS

### Overview of sulfur oxidation pathways in the Bohai Sea sediments

We sequenced metagenomes from 15 sediment samples with an average of 932.08 ± 51.51 million reads per sample (Tables S1 and S2). About half of the raw reads (43.77% ± 4.48%) were assembled into scaffolds greater than 2,000 bp (Table S2). We further recovered 5,233 metagenome-assembled genomes (MAGs) with completeness of over 50% and contamination of less than 10%. A total of 30.45% ± 2.81% raw reads were binned into the MAGs (Table S2). The high proportion of unbinned reads suggests a large unrecovered microbial diversity and the need for further studying the microbial driving biogeochemical cycling, including sulfur oxidation, in coastal marine sediments.

**Fig 1:**
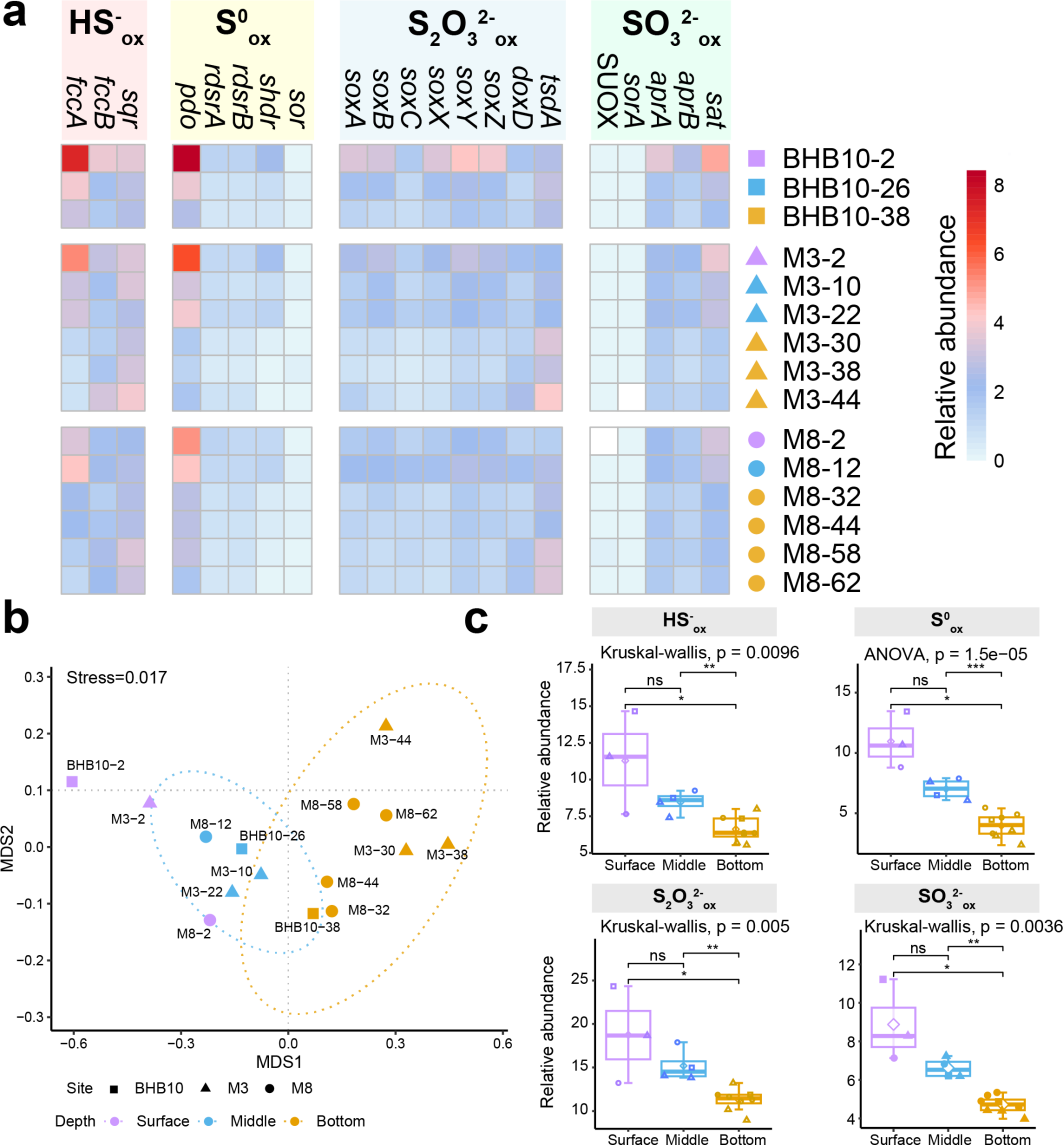
Composition of sulfur oxidation genes in Bohai Sea sediments. (**a**) Heatmap of the normalized relative abundance of genes oxidizing different types of reduced sulfur in different depths at three sampling stations (M3, M8, and BHB10). (**b**) NMDS analysis based on the relative abundance of different genes related to sulfur oxidation in different depths at three sampling stations with 95% confidence shown with dashed ellipses. (**c**) Sum of relative abundance of different genes based on pathways oxidizing different reduced sulfur in three layers (surface, middle, and bottom) at three stations. Surface layer (0–2 cm): samples BHB10-2, M3-2, and M8-2. Middle layer (8–30 cm): samples BHB10-26, M3-10, M3-22, and M8-12. Bottom layer (below 30 cm): samples BHB10-38, M3-30, M3-38, M3-44, M8-32, M8-44, M8-58, and M8-62.

Sulfur oxidation genes involved in different processes were prevalent across all samples (Fig. 1a; Table S3). Genes involved in various pathways for sulfur oxidation showed distinct compositions in surface, middle, and bottom layers (Fig. 1b). The compositions of the sulfur oxidation genes in 0–2 cm samples (M3-2, M8-2, and BHB10-2) differed from the rest of the samples (Fig. 1b). Thus, M3-2, M8-2, and BHB10-2 were classified as surface layer samples. Samples from 8 to 30 cm and below 30 cm were classified as middle and bottom layer samples, respectively. Sulfide-, zerovalent sulfur-, thiosulfate-, and sulfite-oxidizing genes were significantly more abundant in the surface layer than in the bottom layer (*P* < 0.05; Fig. 1c). *sqr*, *pdo*, and *soxABCXYZ* were the dominant genes for sulfide, zerovalent sulfur, and thiosulfate oxidation, respectively, with a tendency of decreased relative abundance with depth (Fig. S1). The *soxD* gene was absent in our samples. The Sox system without SoxD only partially oxidizes thiosulfate, producing sulfate and zerovalent sulfur (41). The relative abundance of *tsdA* (32), which encodes TsdA oxidizing thiosulfate to tetrathionate, tends to increase from the surface to the bottom. The absence of annotated TetH suggests that tetrathionate was likely converted by SoxB in Bohai Sea sediments. The indirect AMP-dependent sulfite oxidation pathway, consisting of oxidative type *aprAB* and *sat* genes (28), was the most abundant among all sulfur-oxidizing genes and decreased with depth (Fig. S1). Surprisingly, the direct sulfite-oxidizing enzymes were scarcely present: genes encoding SUOX and the periplasmic heterodimeric SorA had a low abundance in most samples, and the membrane-bound SoeABC was not detected in all samples.

### Heterotrophy dominates sulfur oxidation in the Bohai Sea sediments

**Fig 2:**
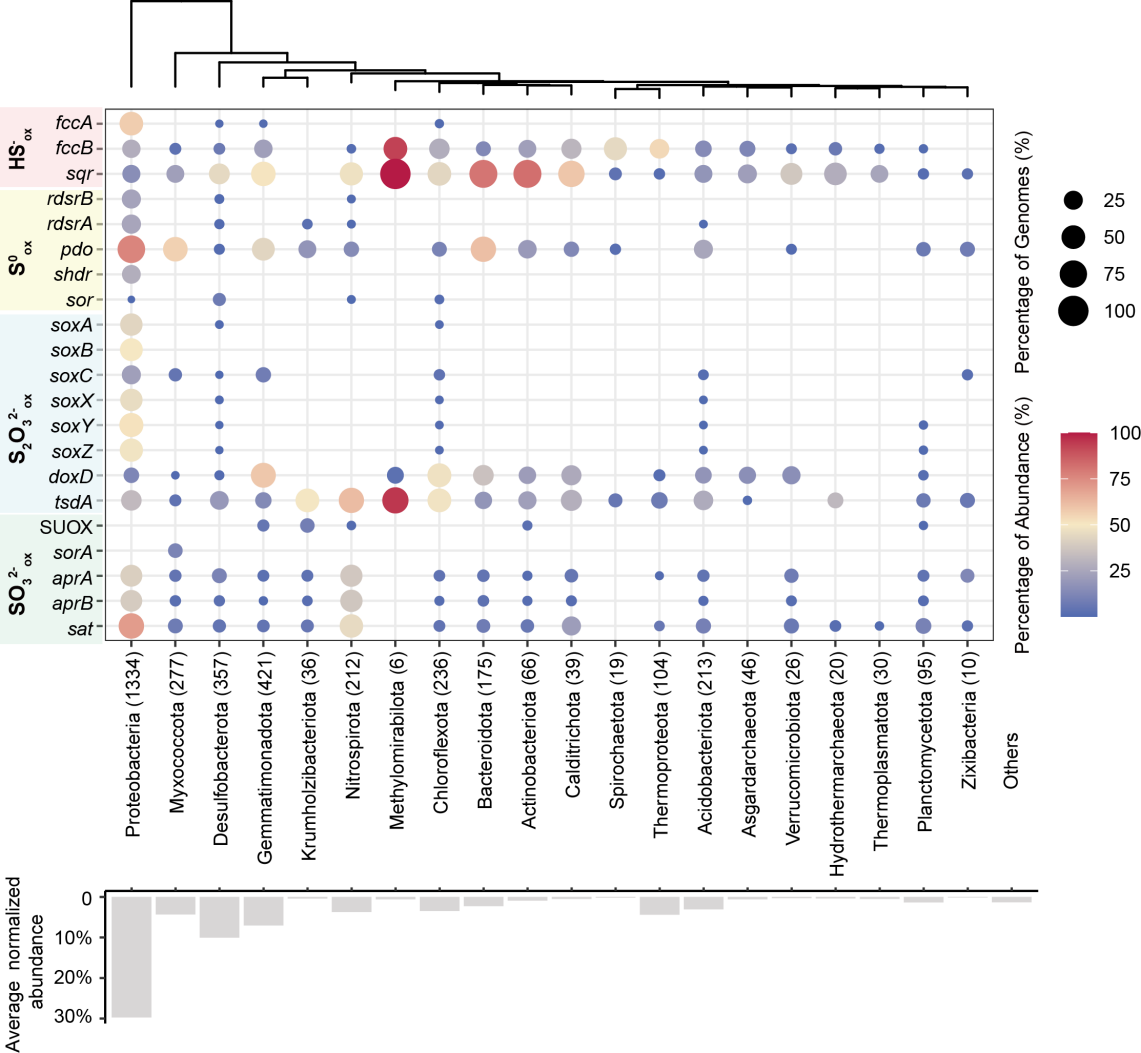
Summarization of MAGs containing sulfur oxidation genes. Colors represent the ratio of MAGs containing sulfur oxidation genes within a phylum based on relative abundance. Sizes the ratio of MAGs containing sulfur oxidation genes within a phylum based on the number of MAGs. Numbers within brackets represent the number of MAGs recovered from 15 sediment samples. Bar plots represent the normalized relative abundance of each phylum.

Sulfur-oxidizing genes were prevalent in these 5,233 MAGs. Over 67.1% of the recovered MAGs (3,511 of 5,233), occupying 67.6% abundance of the recovered community, contain at least one protein sequence related to inorganic sulfur oxidation (sulfide, zerovalent sulfur, thiosulfate, and sulfite; Fig. 2; Fig. S2). Specifically, 32.5% recovered MAGs (1,701 MAGs) contained genes that encode SQR and FCSD for sulfide oxidation; 38.6% had PDO (1,818 MAGs), rDsrAB (350 MAGs), sHdr (329 MAGs), and SOR (39 MAGs) for the oxidation of zerovalent sulfur; 39.7% carried the SoxABXYZ (473 MAGs), TsdA (1,229 MAGs), and DoxD (786 MAGs) for thiosulfate metabolism, and 12.1% possessed complete Sat-AprAB (567 MAGs), SorA (37 MAGs), and SOUX (32 MAGs) for potential sulfite oxidation. These data suggest that biological sulfur oxidation is prevalent in marine sediments (6). These 3,511 MAGs were assigned to 39 bacterial and 5 archaeal phyla, dominated by Proteobacteria, Desulfobacterota, Gemmatimonadota, Myxococcota, and Chloroflexota (Fig. 2).

Heterotrophic and mixotrophic metabolisms dominate the microbial community in the Bohai Sea sediments. We identified 755 MAGs with autotrophic metabolisms (Wood-Ljungdahl pathway, Calvin-Benson-Bassham, reductive tricarboxylic acid, 3-hydroxypropionate bicycle, 3-hydroxypropionate-4-hydroxybutyrate, dicarboxylate-4-hydroxybutyrate cycles, and reverse glycine cleavage pathway; Tables S4 and S5). They mostly belong to Proteobacteria (264 MAGs), Desulfobacterota (125 MAGs), and Nitrospirota (72 MAGs). The remaining 4,478 MAGs, occupying 85.57% of the entire community, likely rely on heterotrophic and mixotrophic metabolisms. They mainly belong to Proteobacteria (1,108 MAGs), Desulfobacterota (432 MAGs), Gemmatimonadota (425 MAGs), and Myxococcota (378 MAGs). A total of 2,954 of the 4,478 MAGs with heterotrophic/mixotrophic metabolism are potentially capable of oxidizing different types of sulfur as evidenced by possessing different sulfur oxidation genes. For example, 1,581 MAGs contain genes encoding SQR (1,220 MAGs) or FccB (the catalytic unit of FCSD; 655 MAGs). A total of 1,702 MAGs encode different enzymes, including PDO (1,571 MAGs), rDsrAB (244 MAGs), sHdr (259 MAGs), and SOR (15 MAGs) that oxidize zerovalent sulfur. Among 1,785 MAGs that oxidize thiosulfate, 1,005 and 740 MAGs oxidize thiosulfate to tetrathionate with TsdA and DoxD, respectively, and 436 MAGs oxidize thiosulfate to sulfate and zerovalent sulfur with the Sox system lacking SoxD. A total of 393 MAGs carry genes encoding Sat-AprAB that oxidize sulfite to sulfate (Table S6).

#### Sulfide oxidation

A total of 2,593 of 3,473 SQR homologous sequences, annotated by the combination of IMG/JGI MAP and diamond search, were confirmed as SQR, and the remaining 880 SQR homologs were confirmed as the catalytic subunit of FCSD (FccB) by their phylogeny (42) (Fig. S3). Phylogenetic analyses suggest that most of these SQR sequences belong to the membrane-bound type III (1,814), type II (378), and type I (296) SQRs, and few of them belong to type IV (9) and type VI (83) SQRs (42). Over half of SQRs (1,707/2,593) were assigned in 1,507 MAGs. Both SQR and FccBs were mainly distributed in Proteobacteria, Gemmatimonadota, and Chloroflexota (Table S7). The significantly higher abundance of genes encoding SQR than FccB in the middle and bottom layers (*P* < 0.01, *t*-test) suggests that SQR was the dominant enzyme oxidizing sulfide in Bohai Sea sediments (Fig. S4).

#### Zerovalent sulfur oxidation

PDOs were the most abundant, followed by sHdr and rDsrABs for zerovalent sulfur oxidation in the sediments. A total of 3,055 PDO sequences were identified from 15 assemblies. A maximum likelihood phylogenetic tree showed that 1,091, 1,150, and 814 PDO sequences were classified as type I, type II, and type III PDOs (43, 44), respectively (Fig. S5). A total of 2,047 of 3,055 identified PDO sequences were binned in 1,818 MAGs. Type I PDOs were mainly identified in Proteobacteria (469 MAGs; 295 Gammaproteobacteria MAGs and 174 Alphaproteobacteria MAGs) and Myxococcota (178 MAGs). Type II PDOs were mainly identified in Proteobacteria (653 MAGs), mostly Gammaproteobacteria (579 MAGs) and Alphaproteobacteria (74 MAGs). Type III PDOs were distributed in more phyla (19 phyla) than type I and type II PDOs, mainly in Gemmatimonadota (191 MAGs) and Bacteroidota (114 MAGs; Table S8). Types I and II PDOs are present only in gram-negative bacteria, while type III PDOs were identified in both gram-negative and positive bacteria but mainly in gram-positive bacteria (43–45). Type II PDOs were commonly distributed in the surface, while types I and III PDOs were often identified in the bottom layer (Fig. S5).

We identified 2,621 α subunit (DsrA) and 2,543 β subunit (DsrB) sequences of dissimilatory sulfite reductase (Dsr), which could be assigned in 1,514 MAGs, in the Bohai Sea sediment samples. The phylogenetic tree showed that 624 DsrA and 601 DsrB sequences are the oxidative bacterial type oxidizing zerovalent sulfur (16) (Fig. S6). rDsrAB sequences were mainly distributed in Gammaproteobacteria (291 MAGs) and Alphaproteobacteria (59 MAGs). Bacterial phyla CG2-30-53-67 (9 MAGs) and Desulfobacterota (6 MAGs), which are mostly considered sulfate reducers, also have rDsrAB. rDsrAB sequences were mainly identified in the surface, while the reductive bacterial type DsrAB sequences were mostly distributed in the bottom layer in the Bohai Sea sediment (Fig. S6). Overall, rDsrAB was identified in 350 MAGs. Of these, 337 MAGs also harbored the gene encoding DsrC. A gene set encoding oxidative-type DsrABCEFH was identified in 307 MAGs, of which 296 MAGs also carried the gene encoding TusA (Table S9).

sHdr is encoded by a *hdrC1B1A-hyp-hdrC2B2* cluster acting as a sulfur-oxidizing entity (18, 19). In total, 406 sHdr were annotated, and 334 of them were assigned to 329 Proteobacteria MAGs (Fig. S7), belonging to Gammaproteobacteria (296 MAGs) and Alphaproteobacteria (33 MAGs). Several orders, including Woeseiales, Kilonielales, UBA8366, and SMXQ01, could oxidize zerovalent sulfur by using sHdr. Gammaproteobacteria containing genes encoding sHdr were mainly identified in the surface sediment, while Alphaproteobacteria harboring genes encoding sHdr were mostly distributed in the bottom layer (Fig. S7).

Only 105 SOR sequences were identified, and 50 of them were assigned in 39 MAGs from 14 samples. A total of 33 of 39 MAGs were classified as Desulfobacterota, which is known for dissimilatory sulfate reduction (46). The narrow distribution of SOR suggests that it is a conserved gene within a small community with rare horizontal gene transfer across different phyla, and its rare presence suggests that it is probably not a key enzyme for zerovalent sulfur oxidation in Bohai Sea sediments.

#### Thiosulfate oxidation

SoxABCXYZ, TsdA, and DoxD (31) were annotated for thiosulfate oxidation in all sediment samples. However, the absence of subunit SoxD in the Sox system suggests that the incomplete Sox system only partially oxidizes thiosulfate to sulfate and zerovalent sulfur instead of the complete oxidation of thiosulfate to two sulfates (41). A total of 473 SoxABXYZ complexes were exclusively distributed in Alphaproteobacteria (93 MAGs) and Gammaproteobacteria (380 MAGs), mainly in the order Woeseiales (137 MAGs; Table S10). Among 473 MAGs containing the Sox complex, 460 MAGs also have at least one pathway for zerovalent sulfur oxidation (Fig. S8), suggesting the zerovalent sulfur generated by the incomplete SOX system would continue to be oxidized to sulfite by zerovalent sulfur oxidases. A total of 1,017 SoxB sequences were annotated, and 695 were assigned in 608 MAGs mainly belonging to Proteobacteria (607 MAGs). Gammaproteobacteria with SoxB were mostly distributed in the surface sediment, while Alphaproteobacteria with SoxB were mainly identified in the bottom layer (Fig. S9). SoxB sequences were identified on 976 scaffolds, and 379 of 976 scaffolds only contained gene encoding SoxB without other Sox-related genes (*soxACXYZ*). At the genome level, 21 MAGs possessed the gene encoding SoxB without the presence of all other Sox genes, and 8 of them contained TsdA sequences, suggesting that these bacteria potentially metabolize thiosulfate to tetrathionate by TsdA and then to zerovalent sulfur and sulfate by SoxB (33). A total of 2,606 TsdA and 1,514 DoxD sequences were identified for thiosulfate oxidation to tetrathionate. TsdAs were mainly distributed in Proteobacteria (436 MAGs), Chloroflexota (156 MAGs), Nitrospirota (143 MAGs), and Desulfobacterota (134 MAGs). DoxDs were mainly identified in Gemmatimonadota (252 MAGs), Proteobacteria (167 MAGs), and Chloroflexota (159 MAGs; Table S7). Since more MAGs contained TsdAs and DoxDs than those carried SoxBs, cross feeding between bacteria was likely in metabolizing thiosulfate. The well-known TetH for tetrathionate oxidation from the chemolithotrophic bacterium Acidithiobacillus ferrooxidans was not identified in our metagenomic sequences (47).

#### Sulfite oxidation

Oxidative-type SAT and AprAB catalyzing sulfite oxidation are abundant in the sediments (Fig. 1a), suggesting that the sediments are an active site for sulfite oxidation. We identified 1,708 oxidative-type SAT and 843 AprAB sequences. A total of 630 MAGs harbored genes encoding oxidative-type AprAB, which are mainly Proteobacteria (482 MAGs) and Nitrospirota (86 MAGs; Fig. 2). A total of 1,207 MAGs harbored genes encoding SAT, which are mainly Proteobacteria (841 MAGs), Nitrospirota (115 MAGs), and Myxococcota (40 MAGs; Table S7). Overall, 10.8% of microorganisms (567 MAGs) harbored genes encoding complete oxidative-type AprAB-SAT pathway in Bohai sediments. We further identified 56 SorA sequences oxidizing sulfite, and 37 SorAs were assigned exclusively in 37 Myxococcota MAGs. Among 66 identified SOUX sequences, 34 sequences were assigned in 32 MAGs, mainly Gemmatimonadota (18 MAGs), which may be involved in sulfite oxidation (Table S7).

### Microbial network for sulfur oxidation

**Fig 3:**
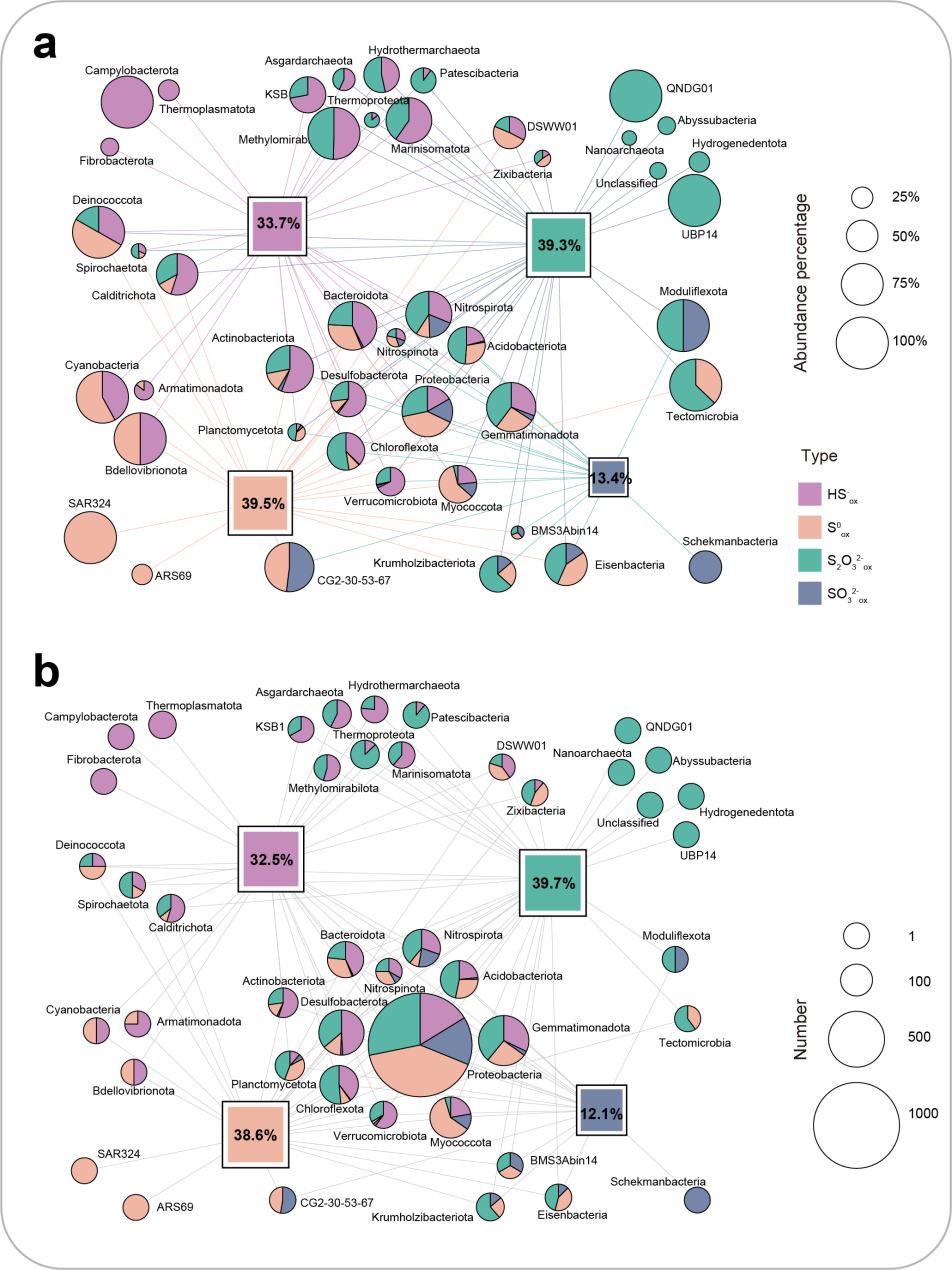
Summarization of different phyla participating in sulfur oxidation. (**a**) Different phyla oxidizing different types of reduced sulfur based on relative abundance. Sizes of squares represent the percentage of MAGs containing genes oxidizing different reduced sulfur in all recovered MAGs based on relative abundance. Sizes of pies represent the percentage of MAGs containing genes oxidizing different reduced sulfur in all recovered MAGs within a single phylum based on relative abundance. (**b**) Different phyla oxidizing different types of reduced sulfur based on the number of MAGs. Sizes of squares represent the percentage of MAGs containing genes oxidizing different reduced sulfur in all recovered MAGs based on counts. Sizes of pies represent the number of MAGs containing genes oxidizing different reduced sulfur in all recovered MAGs within a single phylum based on counts. Colors in line, square, and pie represent four types of reduced sulfur: purple (sulfide), orange (zerovalent sulfur), green (thiosulfate), and cyan (sulfite).

**Fig 4:**
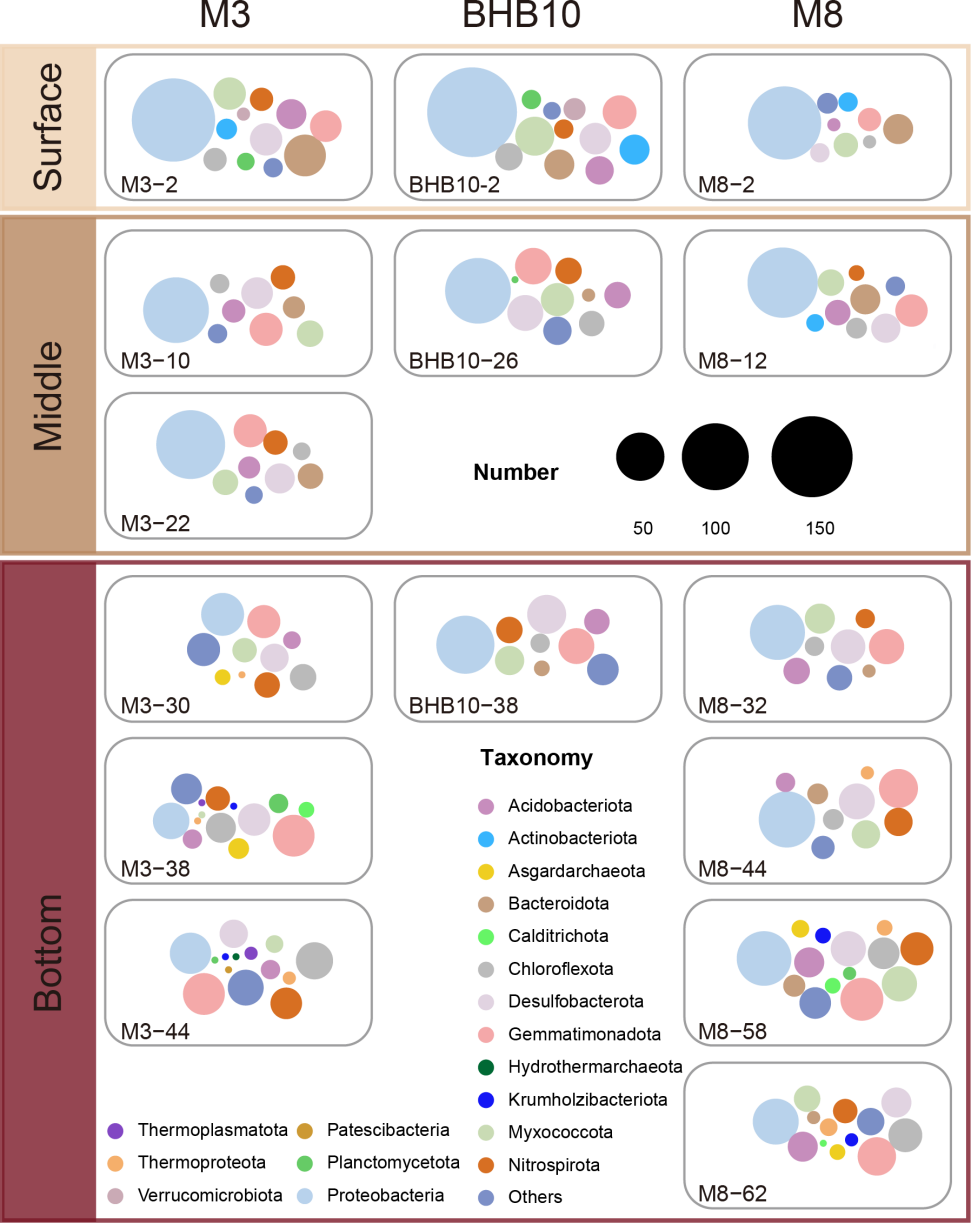
Summarization of different phyla containing sulfur oxidation genes in different layers (surface, middle, and bottom) at three stations (M3, M8, and BHB10). Colors in circles represent different phyla. Sizes represent the number of MAGs.

The dominant phyla involved in sulfur oxidation mainly included Proteobacteria (1,297/1,372 MAGs), Gemmatimonadota (405/438 MAGs), Desulfobacterota (322/564 MAGs), Myxococcota (255/390 MAGs), and Chloroflexota (211/312 MAGs; Fig. 3; Table S11). Abundant lineages participated in the oxidation of multiple sulfur species, while rare taxa tended to only oxidize one species (Fig. 3). Proteobacteria were the dominant group that oxidized different inorganic sulfur species across depths (Fig. 4). However, more phyla were involved in sulfur oxidation in the deeper sediments than in the shallower sediments, and the portion of Proteobacteria for sulfur oxidation decreased with depth (Fig. 4). Desulfobacterota, previously known for dissimilatory sulfate reduction (46) as part of Deltaproteobacteria within the phylum Proteobacteria but recently renamed as a new phylum, also had diverse sulfur oxidation genes. Most Desulfobacterota used SQR for sulfide oxidation and TsdA for thiosulfate oxidation, but many of them lacked the genes for zerovalent sulfur and sulfite oxidation (Fig. 2). Interestingly, Nitrospirota, known as a nitrifying phylum (48–50), also had diverse enzymes for sulfur oxidation, including SQR for sulfide oxidation, rDsrAB, PDO, or SOR for the oxidation of zerovalent sulfur, AprAB and SAT for sulfite oxidation, and TsdA for thiosulfate oxidation. Most Myxococcota (216/390 MAGs), another new phylum originally assigned to Deltaproteobacteria (51, 52), also were abundant in Bohai Sea sediments, and they contained mainly PDO for zerovalent sulfur oxidation. The less abundant enzymes oxidizing sulfite were conservatively distributed in specific lineages, such as Sor sequences being only present in Myxococcota and SUOX sequences being mostly identified in Gemmatimonadota.

### Sulfur species in the sediments

The sulfate concentrations were around 20–25 mM in sediment porewater from 0 to 60 cm at stations M3, M8, and BHB10 (38). The concentration of thiosulfate, sulfide, and sulfite was extremely low (<5 nmol/g sediment) *in situ* in the sediment porewater at station BHB10 (Fig. S10). The maximal concentration of zerovalent sulfur was around 2 µmol/g wet sediment at station M8 and mostly below 1 µmol/g sediment at three stations (~2 mM zerovalent sulfur; Fig. S11). The low concentration of sulfide (<5 µM) and ~2 mM zerovalent sulfur in sediments suggests a high rate of sulfur oxidation in sediments. Alternatively, sulfate is likely reduced by sulfur-reducing bacteria to zerovalent sulfur instead of sulfide (53).

### Estimation of the maximal potential of sulfur oxidation in the sediments

**Fig 5:**
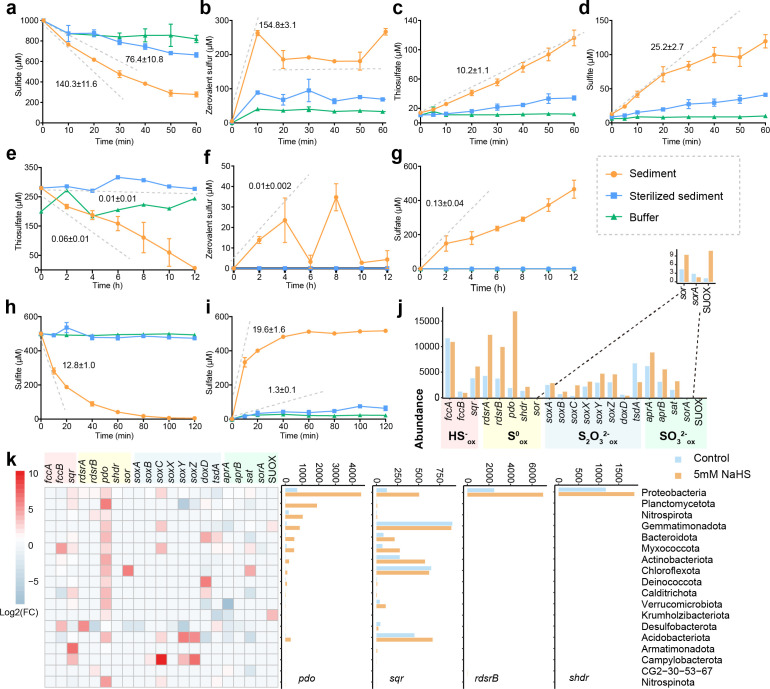
Estimation of sulfur oxidation pathways in sediments. Time course of the concentrations of sulfide (**a**), zerovalent sulfur (**b**), thiosulfate (**c**), and sulfite (**d**) during the incubation at 25°C on a shaking dry bath (1,300 rpm) with the addition of freshly prepared NaHS solution to the final concentration of 1 mmol/L to about 1 g sediments, which was diluted for 100 times with dilution buffer (**a–d**). The maximum bulk and chemical consumption rate of sulfide were 140.58 ± 11.59 and 76.52 ± 10.86 µmol/h/g sediment, respectively (**a**). The maximum production rates for zerovalent sulfur (**b**), thiosulfate (**c**), and sulfite (**d**) were 155.07 ± 3.06, 10.21 ± 1.08, and 25.22 ± 2.69 µmol/h/g sediment, respectively. The production and consumption rates of zerovalent sulfur were balanced after around 10 minutes as shown by the horizontal dashed line (**b**). Time course of the concentrations of thiosulfate (**e**), zerovalent sulfur (**f**), and sulfate (**g**) during the incubation at 25°C on a shaker (200 rpm) with the addition of thiosulfate solution to the final concentration of 200 µmol/L to the system consisting of 20 mL HEPES and about 20 g sediments, which was washed by 20 mL HEPES for five times to remove sulfate in sediment prior to incubation (**e–g**). The maximum bulk consumption rate of thiosulfate (**e**) was 0.06 ± 0.01 µmol/h/g sediment, and the maximum production rates of zerovalent sulfur (**f**) and sulfate (**g**) were 0.01 ± 0.002 and 0.15 ± 0.05 µmol/h/g sediment, respectively. Sulfite was not detected during the incubation with thiosulfate. Time course of the concentrations of sulfite (**h**) and sulfate (**i**) during the incubation at 25°C on a shaker (200 rpm) with the addition of sulfite solution to the final concentration of 500 µmol/L to the system consisting of 9 mL HEPES and about 1 g sediments, which was washed by 9 mL HEPES to remove sulfate in sediment prior to incubation (**h–i**). The maximum consumption rate of sulfite was 13.10 ± 1.05 µmol/h/g sediment (**e**), and the maximum production rate of sulfate was 19.97 ± 1.67 µmol/h/g sediment (**f**). The maximum chemical oxidation rate of sulfite was 1.34 ± 0.15 µmol/h/g sediment based on the production rate of sulfate in sterilized sediment (**f**). Zerovalent sulfur and thiosulfate were not detected during the incubation with sulfite. The dilution buffer without sediment was considered as the blank. Overall relative abundance of gene transcripts oxidizing sulfide, zerovalent sulfur, thiosulfate, and sulfite was higher in the sample with addition of NaHS than in the sample without addition of NaHS (**j**). Gene transcripts of sulfur oxidation pathways were upregulated with addition of NaHS in the top 18 phyla (**k**).

**Fig 6:**
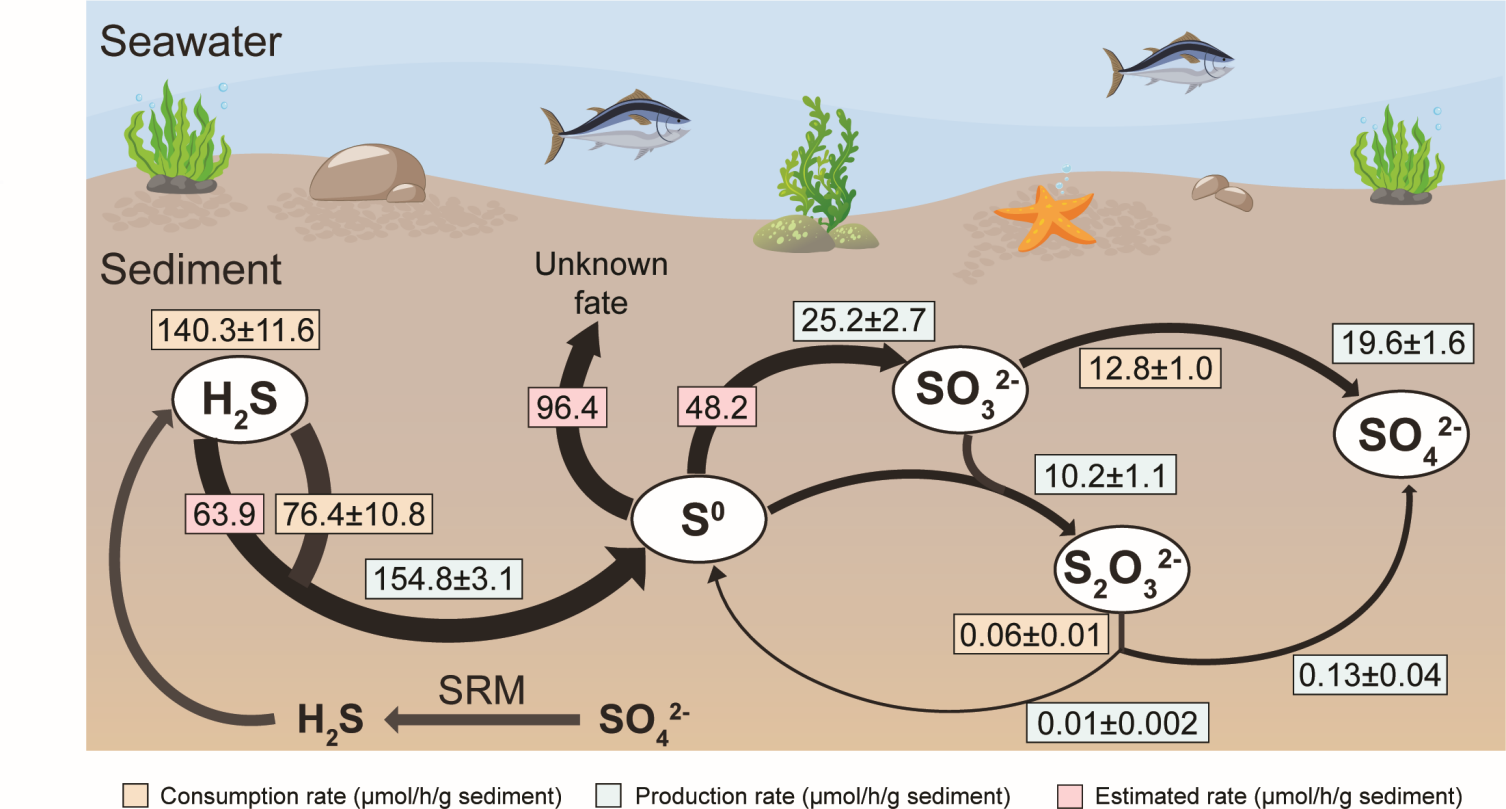
The schematic diagram of maximum sulfur oxidation rate in Bohai surface sediments. The schematic diagram illustrates the measured or estimated maximum consumption rates and maximum production rates of sulfur in different valence states in Bohai Sea surface sediments. The estimated biological sulfide oxidation rate, unknown consumption rate of zerovalent sulfur, and rate of zerovalent sulfur oxidation to sulfite were 64.06, 96.33, and 48.53 µmol/h/g sediment, respectively, shown with the pink background.

Three types of reduced sulfur species (sulfide, thiosulfate, and sulfite) were added to sediment samples to estimate the maximal rates of their oxidation under oxic conditions (Fig. 5). When NaHS was added, sulfide was quickly consumed with maximal rates at 140.3 ± 11.6 and 76.4 ± 10.8 µmol/h/g sediment for biological and chemical consumption, respectively (Fig. 5a). Chemical consumption was estimated by using heat-killed sediments. The maximal production rates of zerovalent sulfur, sulfite, and thiosulfate reached 154.8 ± 3.1, 25.2 ± 2.7, and 10.2 ± 1.1 µmol/h/g sediment during the incubation (Fig. 5b through d). When sulfite was the substrate, the maximal rates of its chemical and biological oxidation were 1.3 ± 0.1 and 12.8 ± 1.0 µmol/h/g sediment (Fig. 5i and h). We washed the sulfate in the sediment before adding thiosulfate to determine the product of thiosulfate oxidation under oxic conditions. When thiosulfate was the substrate, the maximal rates of its chemical and biological oxidation were 0.01 ± 0.01 and 0.05 ± 0.01 µmol/h/g sediment (Fig. 5e). Zerovalent sulfur was not directly tested as the substrate due to its low solubility in water; its maximal rate of oxidation was estimated from sulfite production during sulfide oxidation (Fig. 6), which was the sum of net sulfite production (25.2 ± 2.7 µmol/h/g sediment; Fig. 5d) and sulfite consumption (thiosulfate production and sulfate production, 10.2 ± 1.1 and 12.8 ± 1.0 µmol/h/g sediment, respectively) at about 48.2 µmol/h/g sediment (Fig. 5c, h and 6). Since the maximal rate of thiosulfate oxidation is significantly lower than other rates (*P* < 0.05, analysis of variance [ANOVA]; Fig. S12), thiosulfate oxidation is unlikely to be a dominant reaction in the sediment.

### Metatranscripts during the incubation with sulfide

With the supplements of NaHS, the transcripts mapped to genes for sulfur oxidation were mostly enriched in the whole community (Fig. 5j). The *sqr* transcripts were enriched with supplements of NaHS (Fig. 5j). The abundance of *fccB* (catalytic subunit of FCSD) and *sqr* genes was similar in the surface sediment at station BHB10 (Fig. S4); however, the transcripts of *sqr* were much more abundant than *fccB* in both control and experimental samples, suggesting the important role of SQR for sulfide oxidation in surface sediments. The upregulated *sqr* transcripts were mainly from Proteobacteria, Actinobacteriota, Acidobacteriota, Myxococcota, Bacteroidota, and Verrucomicrobiota (Fig. 5k).

The expression of all genes responsible for zerovalent sulfur oxidation, including *rdsrAB*, *pdo*, *shdr*, and *sor*, was highly upregulated during the incubation (Fig. 5j). The upregulated expression of *rdsrAB* was in a candidate phylum CG2-30-53-67 that consists of known sulfate reducers (16) and different orders belonging to the classes Alphaproteobacteria and Gammaproteobacteria (Fig. S13), including GCA-001735895, Thiohalobacterales, SZUA-229, QNFN01, Woeseiales, and UBA8366 (Fig. S14). The abundance of *pdo* transcripts increased over 76 times in Planctomycetota after adding NaHS (Fig. 5k), suggesting the key role of *pdo* for sulfur oxidation. Twenty phyla, including Proteobacteria (Alphaproteobacteria and Gammaproteobacteria), Nitrospirota, Gemmatimonadota, Bacteroidota, Myxococcota, Acidobacteriota, Actinobacteriota, Chloroflexota, Deinococcota, and Calditrichota (Fig. 5k; Fig. S13), increased the gene expression of *pdo* with the supplements of NaHS, suggesting that the oxidation of zerovalent sulfur is a common function in microbial communities.

The transcripts of the genes encoding the SOX system were slightly enriched during the incubation, mainly due to their expression in Proteobacteria and Campylobacterota (Fig. S15), a chemolithoautotrophic phylum for disproportionation of sulfur previously reported in deep-sea environments (54, 55). Transcripts of *aprAB-sat* for sulfite oxidation increased with the supplements of NaHS, especially in Proteobacteria and Myxococcota, but decreased in Desulfobacterota and Acidobacteriota (Fig. 5k; Fig. S15). The shifts of *aprAB* transcripts among different phyla suggest different responses to NaHS for sulfite oxidation in sediments.

**Fig 7:**
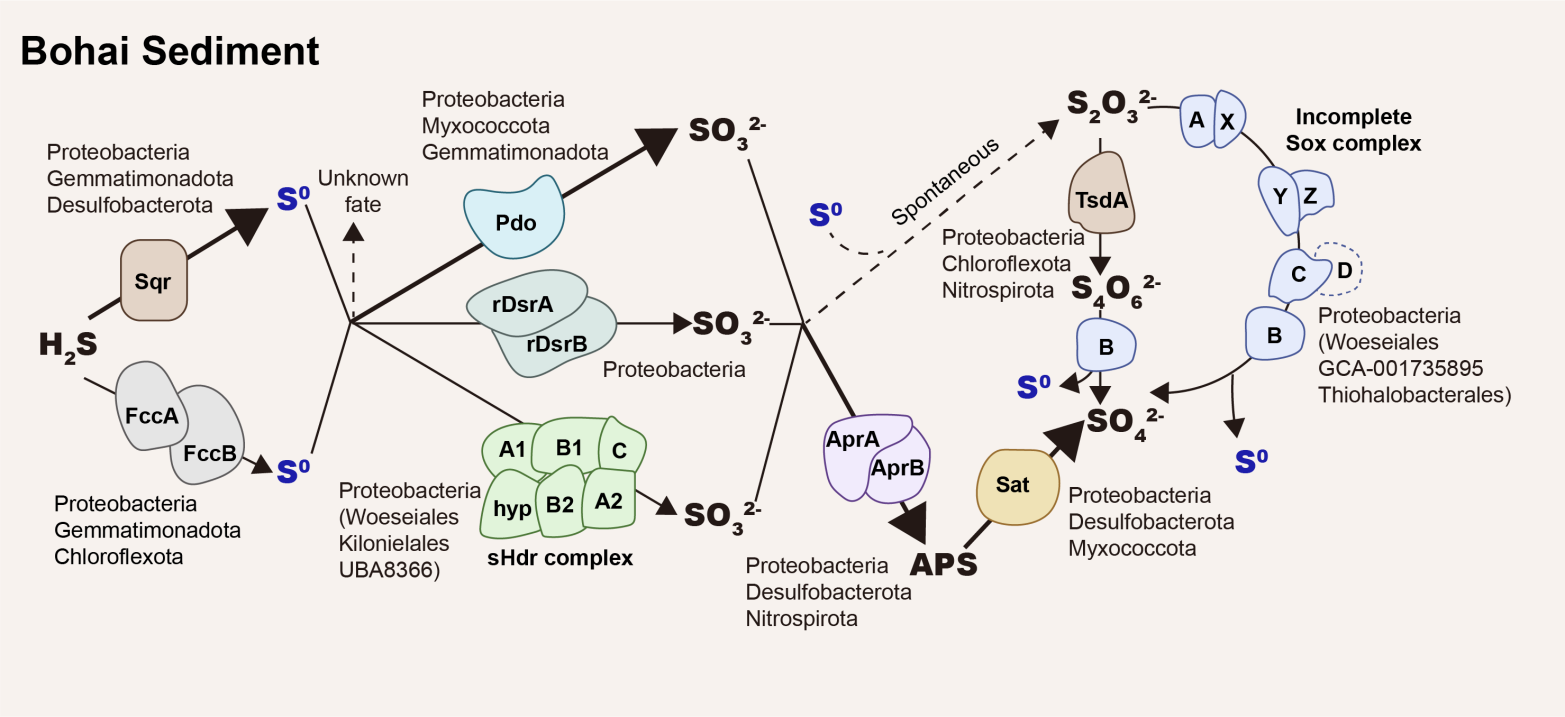
Summarization of the genes, pathways, and microorganisms involved in sulfur oxidation in the Bohai Sea sediments. Sulfide was mainly oxidized by SQR to zerovalent sulfur and further oxidized by PDO, oxidative bacterial-type rDsrAB, and heterodisulfide reductase (Hdr)-like enzyme (sHdr complex) to sulfite. Sulfite was oxidized by AprAB and SAT to sulfate. The oxidation pathway with thiosulfate as the intermediate was not the dominant pathway in the Bohai Sea sediment.

Overall, sulfide is mainly oxidized to zerovalent sulfur by SQR, as the abundance of *sqr* transcripts is much higher than that of *fccB* transcripts in surface sediments (Fig. 5j). Zerovalent sulfur is jointly oxidized to sulfite by PDO, rDsrAB, and sHdr (Fig. 7). *pdo* genes were prevalent among different taxa with much higher relative abundance than *rdsrAB* and *shdr* (Fig. 1 and 2). After the spike of sulfide, the upregulation of *pdo* transcripts was much higher than that of *rdsrAB* transcripts. However, the abundance of *rdsrAB* transcripts was higher than *pdo* and *shdr* transcripts without sulfide addition (Fig. 5j). Sulfite is oxidized to sulfate by the Sat-AprAB pathway. The relative abundances of oxidative type *sat* and *aprAB* genes were higher than other sulfur-oxidizing genes in the sediment (Fig. 5j) (28).

## DISCUSSION

The Bohai Sea is a semi-enclosed continental shelf sea with many aquacultures and a large amount of terrestrial inputs, including nutrients and organic matter (56–58). The zonation in sediment facilitates different biogeochemical processes in different layers. Based on the abundance of marker genes, sulfur oxidation processes primarily occur in surface sediments. Hydrogen sulfide produced from dissimilatory sulfate reduction diffuses to the upper oxygenated zone and is oxidized. Oxygen is the major final electron acceptor in the surface sediment. In the suboxic zone, different oxidized nitrogen compounds, e.g., nitrate and nitrite, could serve as the electron acceptor for sulfur oxidation, which is the denitrification and dissimilatory nitrate reduction to ammonium (DNRA)-coupled sulfur oxidation (59–62). When oxygen and oxidized nitrogen were used up, ferric iron or other oxidized compounds, e.g., arsenate, could thermodynamically serve as the electron acceptor for sulfur oxidation in anoxic environments (63–65). We identified different marker genes involved in denitrification, DNRA, ferric reduction, and arsenate reduction in this study (Table S12), suggesting the coupled sulfur oxidation in sediments. The alternative electron acceptors other than oxygen may play more important roles in Bohai Sea sediments, where the seasonal hypoxia was reported in bottom water, and the rate of mineralization of organic matter was high, resulting in shallow penetration of oxygen in the sediment (66, 67).

The organic-rich sediments provide an active site for microbes to participate in biogeochemical cycling, in which sulfur-oxidizing genes are prevalent in heterotrophic bacteria, suggesting they considerably contribute to sulfur oxidation in sediments. Proteobacteria, especially the classes Alphaproteobacteria and Gammaproteobacteria, are the dominant lineage for sulfur oxidation both in the relative abundance of functional genes and at transcriptomic levels (Fig. 5j; Fig. S13). Sulfur-oxidizing taxa in Bohai Sea sediments are comparable to those in other environments. For instance, the family Rhodobacteraceae within Alphaproteobacteria and the genus *Halomonas* within Gammaproteobacteria harbor genes encoding various enzymes involved in sulfur oxidation in Ney Springs (68). Sulfur-oxidizing bacteria predominantly belong to the class Gammaproteobacteria on the sediment-contacting bottom surfaces of carbonate rocks in the Del Mar East Methane Seep region (69). Cable bacteria played a crucial role in sulfur oxidation in different marine sediments, such as in the Aarhus Bay, North Sea, and Dutch Wadden Sea (70). However, cable bacteria were not detected through metagenomics in sediments from the Bohai Sea and the Yellow Sea (9). Previous studies stressed the importance of chemolithoautotrophic bacteria, such as the Gammaproteobacteria and Epsilonproteobacteria, in sulfide oxidation in marine ecosystems (71–74). Here, we present evidence that heterotrophic bacteria are important for sulfur oxidation, consistent with previous studies in different natural environments, such as Ney Springs (68), Yellow Sea sediments (9), and estuarine sediments in the Songhua River (75). Heterotrophic sulfur oxidation offers a flexible, energy-efficient strategy for microbes in marine sediments, enabling survival in dynamic redox environments while contributing to key biogeochemical processes. The prevalence of Proteobacteria among sulfur oxidation communities decreased with depth, with increased microbial taxa involved in sulfur oxidation, including rare taxa, which often contain genes for specific steps of sulfur oxidation (Fig. 3). The incomplete pathway for sulfur oxidation from sulfide to sulfate within a single genome supports the common handoff of sulfur oxidation within the microbial community and shortcuts in the sulfur cycling (11, 76), which may explain the diverse sulfur-oxidizing groups. The increased microbial interactions are also supported based on the co-occurrence analysis of 16S rRNA genes in the same region (38).

The estimated sulfide oxidation rate in the undiluted marine sediment was 150 nmol/cm^3^/day in Aarhus Bay (77), which is four orders of magnitude lower than our estimated maximal sulfide oxidation rate, suggesting a high potential of sulfur oxidation in Bohai Sea sediments. Additionally, the low *in situ* concentration of sulfide (<0.63 nmol/g sediments) also supports that sulfur oxidation is a general metabolic process in coastal sediments. In contrast, sulfate reduction is a more specialized trait driven by sulfate reducers. Thus, the rate-limiting step of sulfur oxidation is sulfate reduction, which is the main source of sulfide in marine sediments. However, the maximal sulfide oxidation rates reported in our study should not be considered as *in situ* sulfide oxidation rates in Bohai Sea sediments. Excessive input of inorganic nitrogen from increased anthropogenic activities led to eutrophication and formation of oxygen-deficient zones in the central region of the Bohai Sea (66), resulting in the intermittent release of sulfide from anoxic benthic sediments into the overlying water column (78). Through their robust sulfur oxidation capacity and sulfide stress tolerance, sulfur-oxidizing microorganisms sequester sediment-derived sulfide generated from sulfate reduction, forming a critical biogeochemical barrier against oceanic sulfide release.

Sulfide is partially oxidized to elemental sulfur when the molar ratio of oxygen and sulfide is 0.5–1.5, and the higher ratio ensures complete sulfide oxidation to sulfate (79, 80). Due to the low oxygen levels in deep marine sediments, the concentration of zerovalent sulfur was higher in deep sediment than in the surface layer with a range of 0.02–4.3 μmol/g sediment (Fig. S11). Our measured zerovalent sulfur concentrations align with published values from geographically distinct regions, including the Yangtze River Estuary (0.3–2 μmol/g sediment), South Yellow Sea (0–0.3 μmol/g sediment), Okinawa Trough (0–0.7 μmol/g sediment) (81), and other locations in the Bohai Sea (0.01–3.95 μmol/g sediment) (82). These sediment cores reveal stratified geochemical profiles with depth-dependent zerovalent sulfur accumulation patterns consistent with those observed in Bohai Sea sediments. Microbial consortia employ intricate enzymatic networks to optimize elemental sulfur oxidation, with individual genomes often encoding complementary pathways to maximize metabolic flexibility. Genomic surveys reveal the predominance of the PDO pathway in diverse taxa, frequently co-occurring with rDsrAB and/or sHdr complexes (Fig. S8). This dual-system architecture effectively bridges elemental sulfur mobilization with central sulfur metabolism. The rDsr pathway, a recently evolved function linked to Earth’s oxygenated environments and vertically inherited by diverse sulfur-oxidizing bacteria, is a cornerstone of sulfur oxidation in nature (83, 84). Additionally, over 400 MAGs with sulfur oxidation pathways simultaneously harbor Sox complexes in this study, suggesting evolutionary selection for integrated thiosulfate-oxidizing capacity alongside elemental sulfur utilization machinery. The integrated pathways represent an evolutionary adaptation to sulfur-rich environments, where metabolic plasticity ensures energy harvesting across fluctuating sulfur speciation and supply.

Thiosulfate is unlikely to be a major intermediate during sulfide cycling in the Bohai Sea sediments. The maximal oxidation rate of added thiosulfate in our sediment samples under oxic conditions is very slow (~0.06 ± 0.01 µmol/h/g sediment; Fig. 5e) compared to the oxidation rates of sulfide, zerovalent sulfur, and sulfite. We further estimated the maximal consumption rates of thiosulfate under oxic conditions without washing remaining sulfate in the sediment. The maximal consumption rates of thiosulfate did not increase (~0.06 µmol/h/g sediment; Fig. S16). Although the Sox system that oxidizes thiosulfate is present, the *soxD* gene is missing in all the samples. The incomplete Sox system will only partially oxidize thiosulfate to sulfate and zerovalent sulfur instead of two sulfate molecules (19). The absence of the complete Sox systems may be partly responsible for the slow thiosulfate oxidation in the Bohai Sea sediments. The addition of NaHS to the sediments did not significantly upregulate the expression of *sox* genes and other thiosulfate-metabolizing genes. This observation is a surprise, as a major portion of sulfide oxidation through thiosulfate has been reported in other marine sediments (9, 76). Thiosulfate could be reduced by thiosulfate reductase (PhsA) to sulfide by sulfur reducers, and in the absence of additional electron donors, sulfur reducers may oxidize sulfide to zerovalent sulfur, which couples with thiosulfate reduction (85). We identified 197 MAGs, mainly Desulfobacterota (107 MAGs) and Gemmatimonadota (33 MAGs), containing both DsrAB and PhsA, and they are likely sulfur reducers. However, the estimated maximal thiosulfate consumption rate under anoxic conditions was about 0.06 µmol/h/g sediment, similar to the consumption rate under oxic conditions (Fig. S16). Our *in situ* measurements of thiosulfate concentrations (1–3 nmol/g sediment; ∼2–5 µM in porewater) align with those reported from diverse marine sediments, including the Black Sea (~1 µM in porewater) and Arabian Sea (~10 µM in porewater) (86, 87). The production rate of thiosulfate was likely overestimated with the spiking of ample sulfide in the sediments (Fig. S10). The importance of the thiosulfate shunt in sulfur cycling may be overlooked as the estimated maximal potential of sulfur oxidation is higher than the *in situ* rate (77). Nonetheless, the microbial community in the Bohai Sea sediments has limited thiosulfate-oxidizing genes.

Despite its high reactivity rendering tetrathionate elusive in marine sediments, this sulfur intermediate has garnered significant attention for its putative role in sedimentary sulfur cycling (88). Thiosulfate serves as the central biogenic precursor for tetrathionate formation. *Pseudomonas bauzanensis* MTCC 12600, isolated from sediments in the eastern Arabian Sea oxygen minimum zone, possesses the gene encoding TsdA for oxidizing thiosulfate to tetrathionate (87). Metagenomic profiling identified microbial consortia harboring genes dominant encoding TsdA and minor encoding DoxD governing the oxidation of thiosulfate to tetrathionate; however, metatranscriptomic profiles did not respond to the spike of sulfide (*tsdA* gene) and even decreased the expression (Fig. 5j). However, the concentrations of thiosulfate and zerovalent sulfur in nanomolar and micromolar ranges, respectively, observed in the field results, as well as the upregulated expression of the *pdo* gene (zerovalent sulfur oxidation) and downregulated expression of the *tsdA* gene (thiosulfate oxidation) indicate that the zerovalent sulfur within the sediments may be preferentially used in the experimental setup, i.e., under oxic condition, masking possible thiosulfate use. Notably, TetH remained undetectable in our study, leading us to postulate the existence of alternative pathways for tetrathionate oxidation, such as via SoxB for tetrathionate hydrolysis to elemental sulfur and sulfate (33, 89).

The Sat-AprAB pathway is mainly responsible for sulfite oxidation in the Bohai Sea sediment (Fig. 6). Previous study indicated that sulfite was oxidized to form sulfate via the reversed sulfate activation and reduction pathway, i.e., the Sat-AprAB pathway in *Desulfovibrio desulfuricans* (59) and other bacteria (90). Several lines of our evidence indicate that the pathway can also be functional in the Bohai Sea sediments. The genes encoding the Sat-AprAB pathway are abundant, and other well-known enzymes for sulfite oxidation, such as the sulfite dehydrogenases (SoeABC and SorAB) (91, 92) and SUOX (24), are rare or absent. Sulfite dehydrogenases (SoeABC) may play a key role in sulfite oxidation in some other environments, such as the Yellow Sea sediments (9), the Siberian soda lake (93), and the Canadian High Arctic saline Lake (94). The overall transcripts of oxidative type *aprAB* and *sat* were upregulated and higher than other sulfite oxidation genes, and the sulfide spike induced the differential regulation of *aprAB* transcripts from different taxa. The high expression of the genes encoding the Sat-AprAB pathway is likely associated with the high biological sulfite oxidation rate in the sediment samples (Fig. 5j, k and h).

In summary, the integrated analysis of metagenomic data, metatranscriptomic data, physiological experiments, and *in situ* biogeochemical characters showed the complex pathways and diverse players for sulfur oxidation in coastal marine sediments (Fig. 7). Our data extend the diversity of sulfur oxidation lineages. Most microorganisms, mainly heterotrophic bacteria and archaea, participate in the oxidation instead of limited chemolithotrophs. Sulfide is quickly oxidized in the sediments. The main oxidation pathway contains three steps: sulfide oxidation (*sqr* and *fccAB*), zerovalent sulfur oxidation (*pdo*, *rdsrAB*, and *shdr*), and sulfite oxidation (*sat*-*aprAB*; Fig. 7). The common participation in sulfur oxidation by most heterotrophic prokaryotes ensures the surface sediment as a sink for sulfide. However, a comprehensive understanding of genuine sulfur oxidation processes in marine sediments must also take into consideration enzyme affinities and kinetics, as well as the availability of substrates. Moreover, sulfur oxidation may vary in other marine sediments. Further studies are necessary to gain a better understanding of sulfur oxidation in marine sediments.

## MATERIALS AND METHODS

### Sample collection

Coastal sediment core samples were collected from three sites (M3, M8, and BHB10) in the Bohai Sea from 18 to 26 August 2018. Sediment samples from station BHB10 were taken from 15 to 27 July 2022 and from 6 to 26 May 2023. Sampling details and biogeochemical background from the cruise in 2018 were reported previously (38) (Tables S1 and S2). DNA was extracted from 15 samples from sediment cores representing the surface, middle, and bottom layers in three sites and subjected to metagenomic sequencing as previously reported in Gong et al. (38). Sediment samples from the cruise in 2022 and 2023 were taken using the same equipment as the cruise in 2018. Sub-samples were taken at 0, 2.5, 5, 10, 15, 20, 25, and 30 cm in the sediment core. The porewater sample was extracted using Rhizon samplers (pore size of 0.15 µm) and stored at −20°C for the analysis of sulfide, thiosulfate, and sulfite at station BHB10 (0–35 cm) during the cruise in 2022. During the cruise in 2023, two methods for the preservation of sediment samples were applied for the measurement of zerovalent sulfur concentrations: (i) about 0.2–0.3 g sediment was added to an Eppendorf tube with 500 µL ethanol and stored at 4°C; (ii) two sediment samples with a weight of about 0.1 g were added to an Eppendorf tube with 500 µL buffer I and buffer II (95), separately, incubated at 95°C for 10 minutes, and stored at −20°C. Buffer I consisted of 100 mM Tris-HCl, 100 µM diethylenetriaminepentaacetic acid (DTPA), 2% Triton X-100, and 1 mM dithiothreitol. Buffer II consisted of 100 mM Tris-HCl, 100 µM DTPA, 2% Triton X-100, and 2 mM sulfite. Two, two, and one sediment cores (0–30 cm) were taken from stations M3, M8, and BHB10, respectively, and were preserved by using the first method as mentioned above. We applied the second method for the preservation on two sediment cores (0–30 cm) taken from the station M3. Surface sediment samples collected from station M3 for incubation in the laboratory were stored at 4°C during the cruise in 2023.

### Metagenome assembly and binning

Metagenomes were processed with the same procedure as previously reported (35). Briefly, sequences were trimmed and quality controlled using Sickle v1.33 (96) and assembled using IDBA-UD v1.0.9 (97). Scaffolds greater than 2,000 bp were binned using the combination of MaxBin v2.2.7 (98), CONCOCT v0.4.0 (99), MetaBAT v2.12.1 (100), and DASTool v1.1.2 (101) as described previously (102). The quality of MAGs was estimated using CheckM v1.0.5 (103) with lineage_wf. MAGs with over 50% completeness and 10% contamination were manually refined using mmgenome2 (104). Taxonomy of each MAG was assigned using the Genome Taxonomy Database (GTDB)-Tk v1.1.1 (105) with release 202.

### Identification of selected sulfur oxidation genes and autotrophy

Scaffolds ≥2,000 bp were submitted to the Integrated Microbial Genomes and Microbiomes (IMG/M) using DOE-JGI Metagenome Annotation Pipeline (MAP v.4) for gene prediction and functional annotation (106). Sulfur oxidation genes were initially identified based on the KEGG Orthology (KO) assignment (Table S3) from IMG. We curated custom databases for SQR, dissimilatory sulfite reductase (DsrAB), and S-sulfosulfanyl-L-cysteine sulfohydrolase (SoxB) as references to further classify the identified genes from IMG. SQR and DsrAB sequences were downloaded based on previously published references (45, 107–111). SoxB sequences were downloaded from FunGene and further reduced redundancy using CD-HIT v4.8.1 (112) with default settings. We collected PDO sequences as the database and classified them into three types (43). PDO homologs in our data sets were identified using DIAMOND v2.0.8 (113) with the parameters: -e 1e-10 --subject-cover 50 -id 50. Homologs of six subunits of heterodisulfide reductase (Hdr)-like enzyme (sHdr) (19), i.e., HdrC1, HdrB1, HdrA, HYP, HdrC2, and HdrB2, were identified using DIAMOND v2.0.8 against each custom database with the parameters: -e 1e-10 --subject-cover 50 -id 40. Only the sequentially ordered subunits of sHdr in a single scaffold were kept for downstream analysis (19). Oxidative type AprAB and Sat sequences were identified using Disco v1.0.0 (114) with the DiSCo.pl and filter_DiSCo.pl scripts.

Autotrophic pathways, including the Wood-Ljungdahl pathway, Calvin-Benson-Bassham, reductive tricarboxylic acid, 3-hydroxypropionate bicycle, 3-hydroxypropionate-4-hydroxybutyrate, dicarboxylate-4-hydroxybutyrate cycles, and reverse glycine cleavage pathway, were identified based on the annotation from IMG. The pathway was confirmed by the two standards: the presence of 70% genes or the presence of marker genes plus over 50% accessory genes (Table S4).

### Phylogeny of selected genes

We selected six sets of protein sequences, i.e., DsrA, DsrB, SoxB, SQR, PDO, and sHdr clusters for further phylogenetic classification. Each set of identified sequences was aligned with the corresponding custom databases using MAFFT v7.450 (115) with default parameters, except for sHdr sequences. The alignments were trimmed using BMGE v1.12 (116) with the setting “-m BLOSUM62 -g 0.5 -b 3” for DsrA and DsrB and trimAl v1.4.rev22 (117) with “-gappyout” option for SoxB, SQR/FCSD, and PDO. All alignments were manually checked to remove the short alignment, and maximum likelihood phylogenetic trees were generated using IQ-TREE v1.6.12 (118) with the parameters: -m MFP -bb 1,000 -bnni -alrt 1,000 -seed 500 -st AA.

The Hdr-like protein cluster is composed of six subunits in the sequential order: HdrC1, HdrB1, HdrA, HYP, HdrC2, and HdrB2 in a single scaffold (18). Each subunit was aligned using six different algorithms including MAFFT v7.450 (115), ClustalW v2.1 (119), DIALIGN v2.2.1 (120), Muscle v3.8.31 (121), Opal v2.1.3 (122), and Kalign v3.3.1 (123) with default settings (124). The alignment with the highest consistency score for each subunit was selected using trimAl v1.2rev59 (117) with the setting “-compareset” and trimmed with the parameters: -automated1 -resoverlap 0.55 -seqoverlap 60. All trimmed alignments were manually checked. Clusters with at least five sequential ordered subunits were concatenated to build a maximum likelihood phylogenetic tree using IQ-TREE v1.6.12 (118) with the parameters: -m MFP -bb 1,000 -bnni -alrt 1,000 -seed 500 -st AA. All final trees were visualized using the Interactive Tree Of Life (iTOL) webtool (125).

### Calculation of the relative abundance of scaffolds, functional genes, and MAGs

The relative abundance of each scaffold was calculated by the number of reads mapped to the scaffold, further normalized by the length of the scaffold and the number of reads in each sample, and multiplied by 10^6^ for readability purposes. The relative abundance of each functional gene was the sum of the relative abundance of its corresponding scaffold. The relative abundance of MAGs in each sample was calculated using CoverM v0.6.1 (126) by mapping raw reads to the 5,233 MAGs with the parameters: -p bwa-mem --min-read-percent-identity 95 --min-read-aligned-percent 95. To better estimate the relative abundance of different groups with sulfur oxidation genes in the assembled communities, the unmapped ratio was removed, and the sum of the relative abundance of each MAG in one sample was normalized to one. Statistical analyses were carried out in R.

### Visualization of pathways of sulfur oxidation

Associations between different sulfur substrates and phyla were visualized using Gephi (127) and R.

### Measurement of sulfide, zerovalent sulfur, thiosulfate, sulfite, and sulfate in sediment

Zerovalent sulfur in sediment samples was analyzed using the methods reported previously (95). Briefly, about 0.1 g sediment samples (sediment stored in buffer I or buffer II) or a 60 µL mixture of ethanol and sediment (sediment stored in ethanol) was added to the double concentrated reaction buffer (95). Fifty microliter of the supernatant was mixed with 5 µL of 25 mM mBBr and incubated in the dark at room temperature for 25 min, and the reaction was stopped by adding 110 µL of a mixture of acetic acid and acetonitrile (vol/vol, 1:9) (95). After centrifugation, the concentration of thiosulfate in the system was measured using high-performance liquid chromatography (HPLC) (LC-20A, Shimadzu, Kyoto, Japan) with a fluorescence detector (RF20A, Shimadzu, Kyoto, Japan) with detection limits of 0.01 µM (95), and the concentration of zerovalent sulfur was calculated. The concentration of sulfide in porewater was measured using the methylene blue method, with detection limits of 0.5 µM (128). Sulfite and thiosulfate in porewater were analyzed using HPLC as previously reported, with detection limits of 0.5 µM for sulfite and 0.1 µM for thiosulfate (29). Sulfate was measured with an ICS-1100 Ion Chromatography system (Dionex Corporation, CA, USA) using 10 mM KOH as the eluent at a flow rate of 1 mL/min with the detection limit of 0.5 µM. Each sample was analyzed three replicate times.

### Incubation with sulfide, thiosulfate, and sulfite

#### Incubation with sulfide

Seawater was filtered with a 0.22 μm-pore-size filter and further autoclaved. Dilution buffer contained 100 µmol/L DTPA in sterilized seawater. One gram of untreated sediment and autoclaved sediment was separately diluted 100 times with the dilution buffer (99 mL) as the experimental and control groups, respectively. The dilution buffer without sediment was considered as the blank. All three groups (experimental group, control, and blank) were incubated at 25°C on a shaking dry bath (1,300 rpm) with the addition of freshly prepared NaHS solution to the final concentration of 1 mmol/L. Samples were taken every 10 minutes to measure the concentration of sulfide, zerovalent sulfur, thiosulfate, and sulfite as described above.

#### Incubation with thiosulfate

Twenty grams of sediment samples was mixed with 20 mL of 4-(2-hydroxyethyl)-1-piperazine ethanesulfonic acid (HEPES) with a concentration of 30 mmol/L and pH 8.0. After centrifuging for 10 minutes at 4,000 × *g*, the supernatant was removed. This procedure was repeated five times to remove the sulfate in the sediment. The removal of sulfate was confirmed by an ICS-1100 Ion Chromatography system. A total of 20 mL of 30 mmol/L HEPES (pH 8.0) was added to the pellet as the experimental group. The control group was prepared with the same procedure using the autoclaved sediment. The blank was the HEPES solution. All three groups (experimental group, control, and blank) were incubated at 25°C on a shaker (200 rpm) with the addition of thiosulfate solution to the final concentration of 200 µmol/L. Samples were taken every 2 hours to measure the concentration of zerovalent sulfur, thiosulfate, sulfite, and sulfate as described above.

To estimate the total consumption rate of thiosulfate, the sterilized seawater was used as the buffer. Five grams of sediments was diluted with 5 mL (1:1), 10 mL (1:2), and 20 mL (1:4) sterilized seawater, respectively. The diluted samples were incubated at 25°C on a shaker (200 rpm) with the addition of thiosulfate solution to the final concentration of 100 µmol/L under oxic and anoxic conditions. Samples were taken at 0, 30, 60, 120, and 180 minutes to measure the concentration of thiosulfate as described above.

#### Incubation with sulfite

Preparation for the experimental group, control, and blank was the same for the incubation with thiosulfate but changing the weight of sediment to 1 g and volume of HEPES to 9 mL for washing and final dilution. All three groups (experimental group, control, and blank) were incubated at 25°C on a shaker (200 rpm) with the addition of sulfite solution to the final concentration of 500 µmol/L. Samples were taken every 20 minutes to measure the concentration of zerovalent sulfur, thiosulfate, sulfite, and sulfate as described above.

#### Quantification of rates

All experiments were conducted with three replicates. The rates calculated for the experimental group correspond to the total consumption rates, whereas those derived from the control group represent the chemical consumption rates. The biological sulfide consumption rate was estimated by subtracting the chemical consumption rate from the total consumption rate. The sampling interval and dilution factor were optimized based on the consumption rate and feasibility of measurement of each sulfur species. Due to either substrate limitation or product accumulation, the consumption or production rates decreased during the incubation; thus, the maximal rate was estimated based on the first sampling time after substrate addition. An exception occurred in the case of thiosulfate accumulation following sulfide spiking, which progressed linearly. For this exception, the production rate was calculated using data from the entire experimental period. The estimated biological consumption rate of zerovalent sulfur was determined by summing the sulfite production rate and the consumption rates of sulfite. The difference between the production of zerovalent sulfur and its known consumption, specifically the conversion to sulfite and thiosulfate, is termed the “unknown fate” of zerovalent sulfur.

### Incubation for metatranscriptomic analysis and metatranscriptomic data analysis

Five grams of sediment samples was diluted with 5 mL sterilized seawater containing 100 µmol DTPA. The experimental group was supplied with 100 µL of 100 mmol/L NaHS solutions five times with an interval of 30 minutes. Both the experimental group and the control group were incubated at 25°C on a shaker (200 rpm). The concentration of zerovalent sulfur in sediment was measured at 10 and 30 minutes after the last addition of NaHS using the method mentioned above. Sediment samples were then frozen in liquid nitrogen and stored at −80°C for RNA extraction. RNA was extracted using the RNAprep Pure Cell/Bacteria Kit (Tiangen). The quality of RNA extraction was checked on an electrophoresis gel and Agilent 2100. rRNA was removed by TIANSeq rRNA Depletion Kit (Tiangen) following the manufacturer’s protocol. Libraries were prepared using Fast RNA-seq Lib Prep Kit V2 (ABclonal). Metatranscriptomic data were sequenced on an Illumina Novaseq X Plus platform. rRNA in raw data from the control and experimental samples was further filtered using SortMeRNA v4.3.6 (129) against rRNA database release 4.3.4 with the parameters: -fastx --paired_out. Cleaned data were mapped to the predicted protein sequences from all 15 assemblies using BWA-MEM v0.7.17 (130) with default settings to generate sam files. The generated sam files were converted to bam files using SAMtools v0.1.19 (131) with parameters: view -bS -F 4 and further sorted using SAMtools v0.1.19 with default parameters. The resulting bam files were summarized using jgi_summarize_bam_contig_depths in MetaBAT v2.12.1 (100) to generate the depth file. The relative abundance was the calculated depth normalized by the total number of cleaned sequences in control and experimental samples and multiplied by 10^9^ for readability purposes. The change of gene expression was calculated by the equation: log2FC = log2 (experimental sample) − log2 (control).

### Statistical analysis

All statistical analyses and graphics were conducted using the R software environment. Non-metric multidimensional scaling (NMDS) ordination, based on Bray-Curtis dissimilarity, was applied to assess sulfur-oxidizing organism community structure using the relative abundance of sulfur oxidation genes (via the vegan package). The relative abundances of sulfur oxidation genes across sediment layers (surface, middle, and bottom) were statistically compared using either ANOVA or the Kruskal-Wallis test. ANOVA was selected when data conformed to a normal distribution; otherwise, the Kruskal-Wallis test was applied. Data visualization was implemented using the ggplot2 and ggpubr packages.

## Data Availability

All sequences and annotations of scaffolds are available at IMG/JGI under GOLD Study ID Gs0149406. Protein sequences of confirmed key enzymes, relevant statistical data, phylogenetic tree, and metatranscriptomic data are available on Figshare (10.6084/m9.figshare.29234873).

## SUPPLEMENTAL MATERIAL

Supplemental figures mbio.01722-25-s0001.pdf Fig. S1–S16.

Supplemental tables mbio.01722-25-s0002.xlsx Tables S1–S12.

ASM does not own the copyrights to Supplemental Material that may be linked to, or accessed through, an article. The authors have granted ASM a non-exclusive, world-wide license to publish the Supplemental Material files. Please contact the corresponding author directly for reuse.
