# Supplementary material for: Overlooked role of heterotrophic prokaryotes in sulfur oxidation makes the sediment of the Bohai Sea a sufficient sink of hydrogen sulfide
Source: mBio. 2025 Jul 7;16(8):e01722-25. doi: 10.1128/mbio.01722-25 (PMC12345210; doi:10.1128/mbio.01722-25)
Supplement: Supplemental figures — Fig. S1–S16. [file mbio.01722-25-s0001.pdf]

Supplementary Information

Overlooked role of heterotrophic prokaryotes in sulfur oxidation makes the sediment of the Bohai Sea a sufficient sink of hydrogen sulfide

Zhiyi Chen[1,2], Luying Xun[1,3], Yongzhen Xia[1]*, Xianzhe Gong[2,4,5]*

1. State Key Laboratory of Microbial Technology, Shandong University, Qingdao, Shandong 266237, China
2. Institute of Marine Science and Technology, Shandong University, Qingdao, Shandong 266237, China
3. School of Molecular Biosciences, Washington State University, Pullman, WA 99164-7520, USA
4. Southern Marine Science and Engineering Guangdong Laboratory (Guangzhou), Guangdong, 511458, China
5. Department of Marine Science, Marine Science Institute, University of Texas at Austin, TX 78373, USA

*Corresponding Authors:

Yongzhen Xia xiayongzhen2002@sdu.edu.cn;

Xianzhe Gong xianzhe.gong@gmail.com

Supplementary Figures

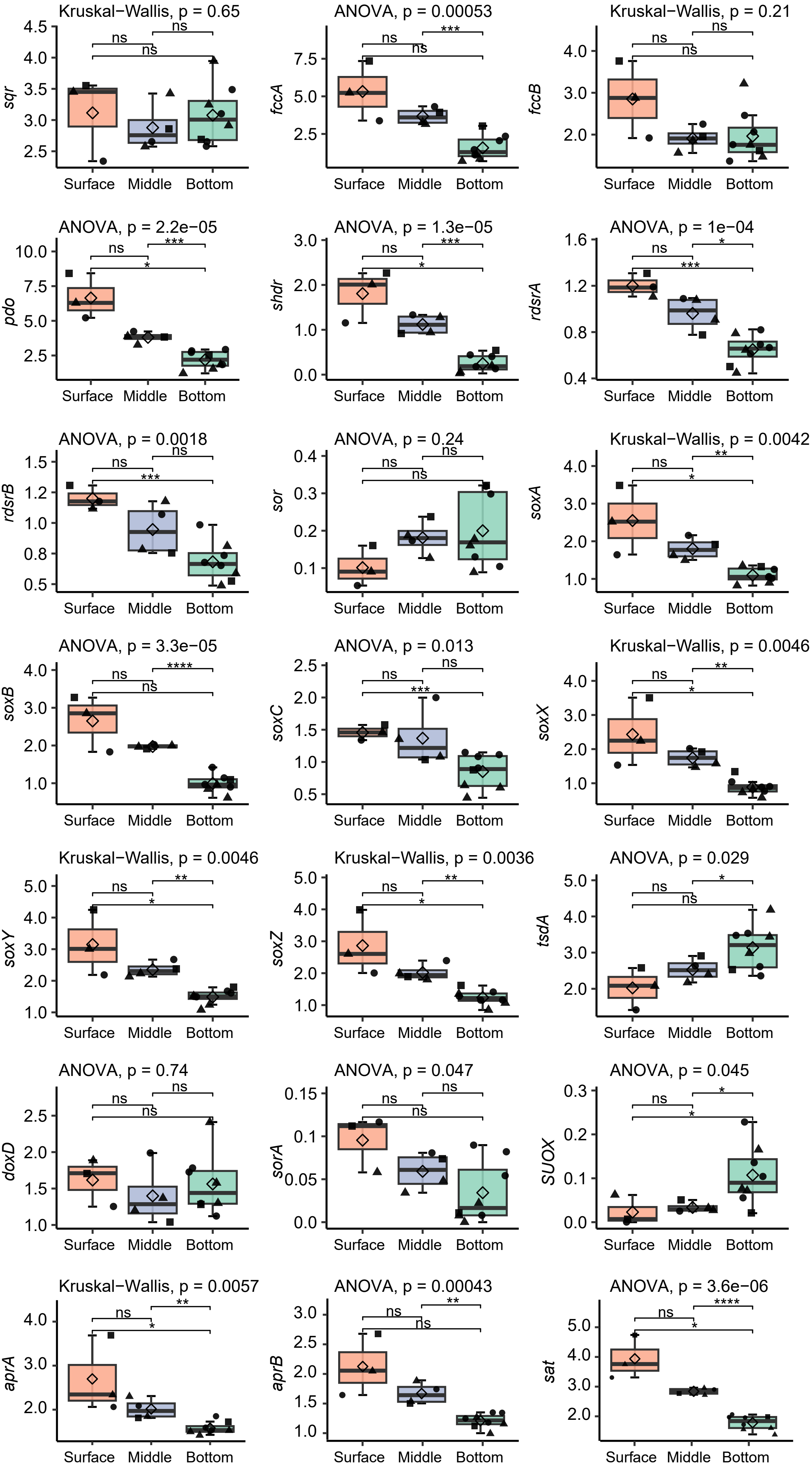

Kruskal−Wallis, p = 0.65
sqr
ns ns ns
ANOVA, p = 0.00053
fccA
ns *** ns
Kruskal−Wallis, p = 0.21
fccB
ns ns ns
ANOVA, p = 2.2e−05
pdo
ns *** *
ANOVA, p = 1.3e−05
shdr
ns *** *
ANOVA, p = 1e−04
rdsrA
ns * ***
ANOVA, p = 0.0018
rdsrB
ns ns ***
ANOVA, p = 0.24
sor
ns ns ns
Kruskal−Wallis, p = 0.0042
soxA
ns ** *
ANOVA, p = 3.3e−05
soxB
ns **** ns
ANOVA, p = 0.013
soxC
ns ns ***
Kruskal−Wallis, p = 0.0046
soxX
ns ** *
Kruskal−Wallis, p = 0.0046
soxY
ns ** *
Kruskal−Wallis, p = 0.0036
soxZ
ns ** *
ANOVA, p = 0.029
tsdA
ns * ns
ANOVA, p = 0.74
doxD
ns ns ns
ANOVA, p = 0.047
sorA
ns ns ns
ANOVA, p = 0.045
SUOX
ns * *
Kruskal−Wallis, p = 0.0057
aprA
ns ** *
ANOVA, p = 0.00043
aprB
ns ** ns
ANOVA, p = 3.6e−06
sat
ns **** *
Surface Middle Bottom

Supplementary Figure 1. Statistical analysis of relative abundance of different sulfur oxidation genes in different layers (surface, middle, and bottom) at three stations (M3, M8, and BHB10).

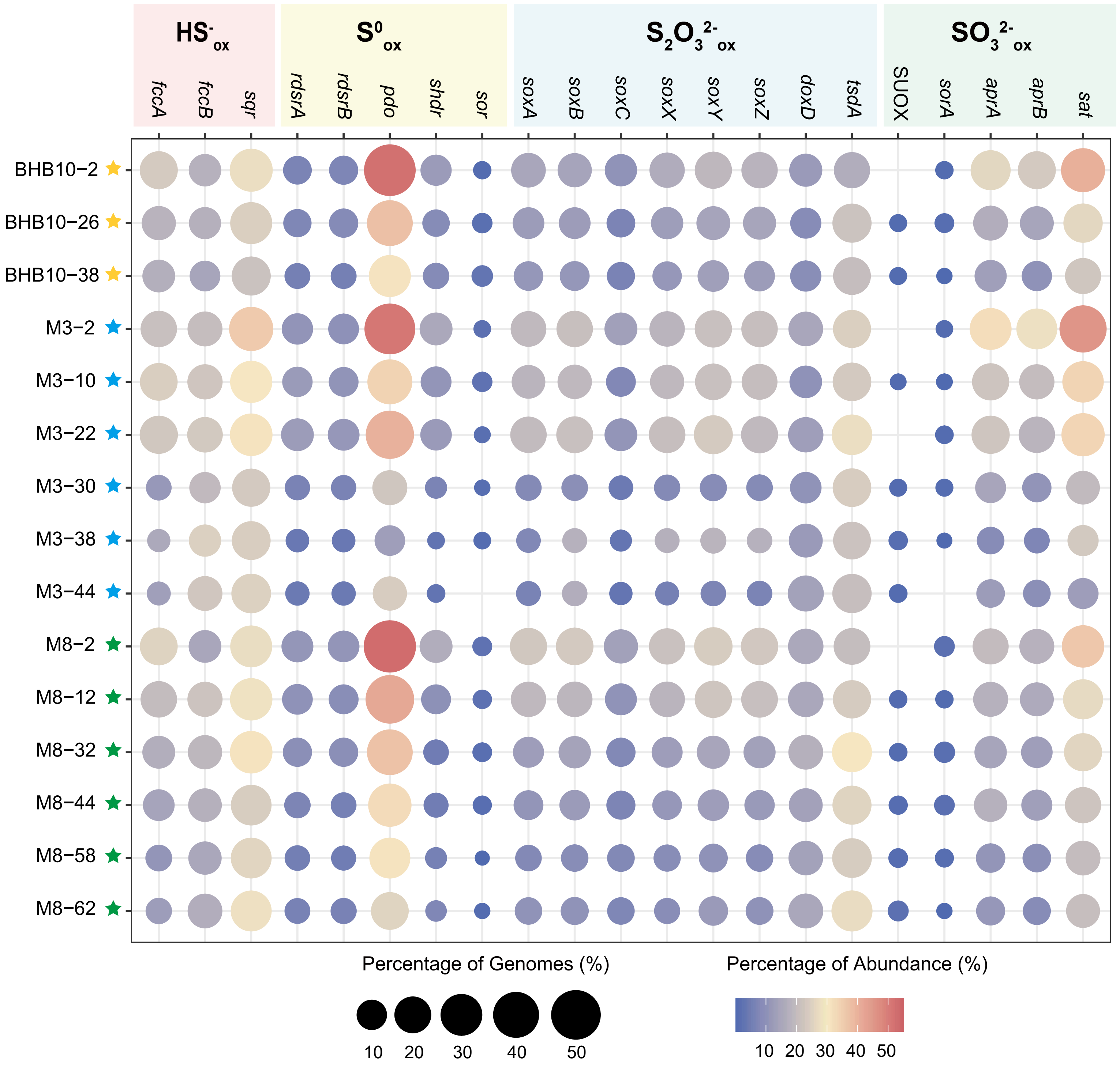

HS⁻ ox
S⁰ ox
S2O3 2- ox
SO3 2- ox
fccA
fccB
sqr
rdsrA
rdsrB
pdo
shdr
sor
soxA
soxB
soxC
soxX
soxY
soxZ
doxD
tsdA
SUOX
sorA
aprA
aprB
sat
BHB10−2
BHB10−26
BHB10−38
M3−2
M3−10
M3−22
M3−30
M3−38
M3−44
M8−2
M8−12
M8−32
M8−44
M8−58
M8−62
Percentage of Genomes (%)
10 20 30 40 50
Percentage of Abundance (%)
10 20 30 40 50

Supplementary Figure 2. Summarization of MAGs containing sulfur oxidation genes in different samples. Sizes represent the percentage of MAGs containing sulfur oxidation genes within one sample based on counts of MAGs. Colors represent the percentage of MAGs containing sulfur oxidation genes within one sample based on relative abundance of MAGs.

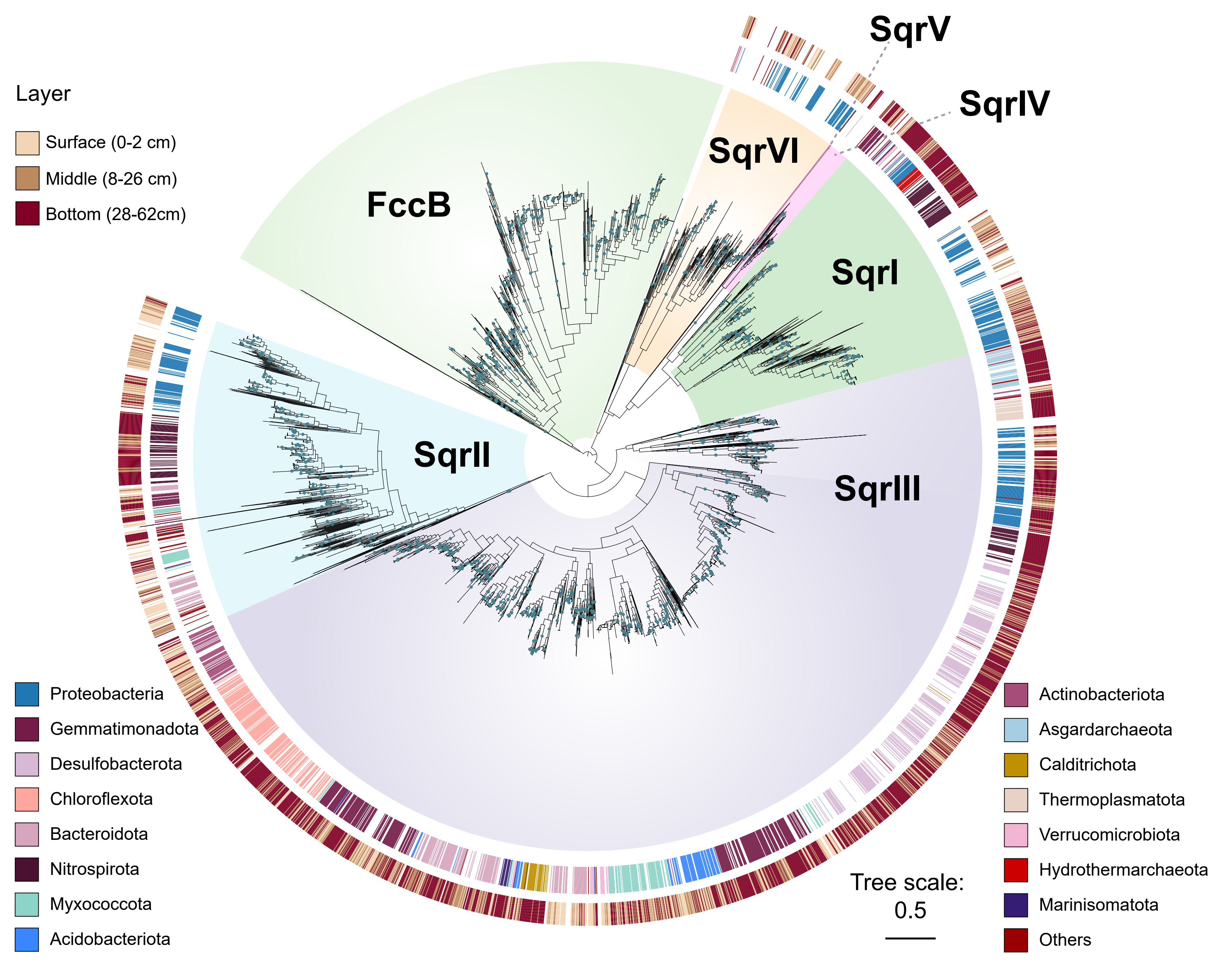

Layer
Surface (0-2 cm)
Middle (8-26 cm)
Bottom (28-62cm)
SqrV
SqrIV
SqrVI
FccB
SqrI
SqrII
SqrIII
Proteobacteria
Gemmatimonadota
Desulfobacterota
Chloroflexota
Bacteroidota
Nitrospirota
Myxococcota
Acidobacteriota
Tree scale:
0.5
Actinobacteriota
Asgardarchaeota
Calditrichota
Thermoplasmatota
Verrucomicrobiota
Hydrothermarchaeota
Marinisomatota
Others

Supplementary Figure 3. Maximum likelihood phylogenetic tree of sulfide:quinone oxidoreductase (SQR) homologous protein sequences, including 3,473 sequences annotated in this study and 1,753 sequences downloaded from publicly available databases as reference. Bootstrap values ≥90 are shown in circles. Sequences were aligned using MAFFT v7.475, trimmed using trimAl v1.4. The phylogenetic tree was constructed using RAxML v8.2.12.

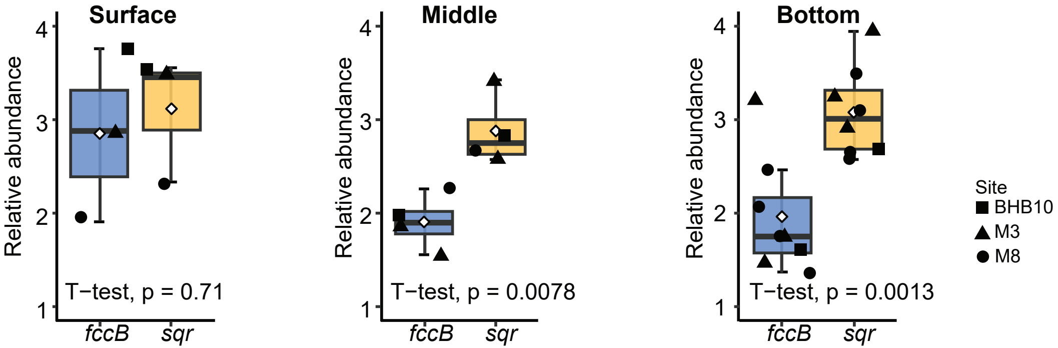
Surface
Middle
Bottom
Relative abundance
1
2
3
4
fccB
sqr
T−test, p = 0.71
T−test, p = 0.0078
T−test, p = 0.0013
Site
BHB10
M3
M8

Supplementary Figure 4. Statistical analysis of relative abundance of sulfide oxidation genes (*sqr* and *fccB*) in three layers (surface, middle, and bottom).

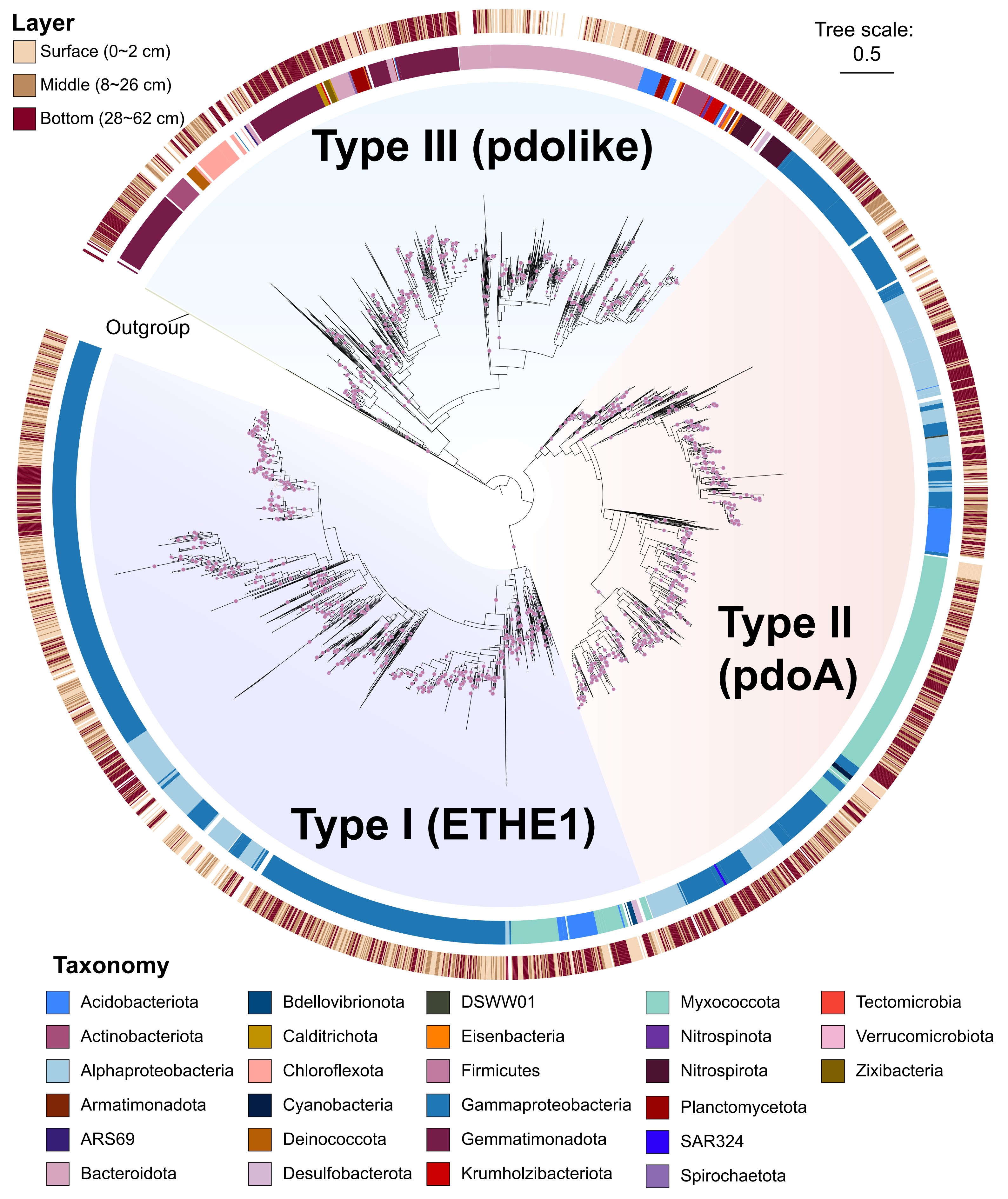
Layer
Surface (0~2 cm)
Middle (8~26 cm)
Bottom (28~62 cm)
Tree scale:
0.5
Type III (pdolike)
Outgroup
Type II
(pdoA)
Type I (ETHE1)
Taxonomy
Acidobacteriota
Actinobacteriota
Alphaproteobacteria
Armatimonadota
ARS69
Bacteroidota
Bdellovibrionota
Calditrichota
Chloroflexota
Cyanobacteria
Deinococcota
Desulfobacterota
DSWW01
Eisenbacteria
Firmicutes
Gammaproteobacteria
Gemmatimonadota
Krumholzibacteriota
Myxococcota
Nitrospinota
Nitrospirota
Planctomycetota
SAR324
Spirochaetota
Tectomicrobia
Verrucomicrobiota
Zixibacteria

Supplementary Figure 5. Phylogeny of persulfide dioxygenase (PDO) protein sequences and distribution of different types of PDOs. (a) A maximum likelihood phylogenetic tree of persulfide dioxygenase (PDO) protein sequences, including 3,055 sequences annotated in this study and 1,920 sequences downloaded from publicly available databases as reference. Ten sequences of glyoxalase were rerooted as the outgroup. Outer ring represents the sampling layer recovering the sequence. Inner ring represents the taxonomy. Bootstrap values ≥90 are shown in circles. Sequences were aligned using MAFFT v7.475, trimmed using BMGE v1.12. The phylogenetic tree was constructed using IQ-TREE v1.6.12. (b) Bar plot showing the percentage of counts of different types of PDO sequences in different samples at three sampling stations (M3, M8, and BHB10).

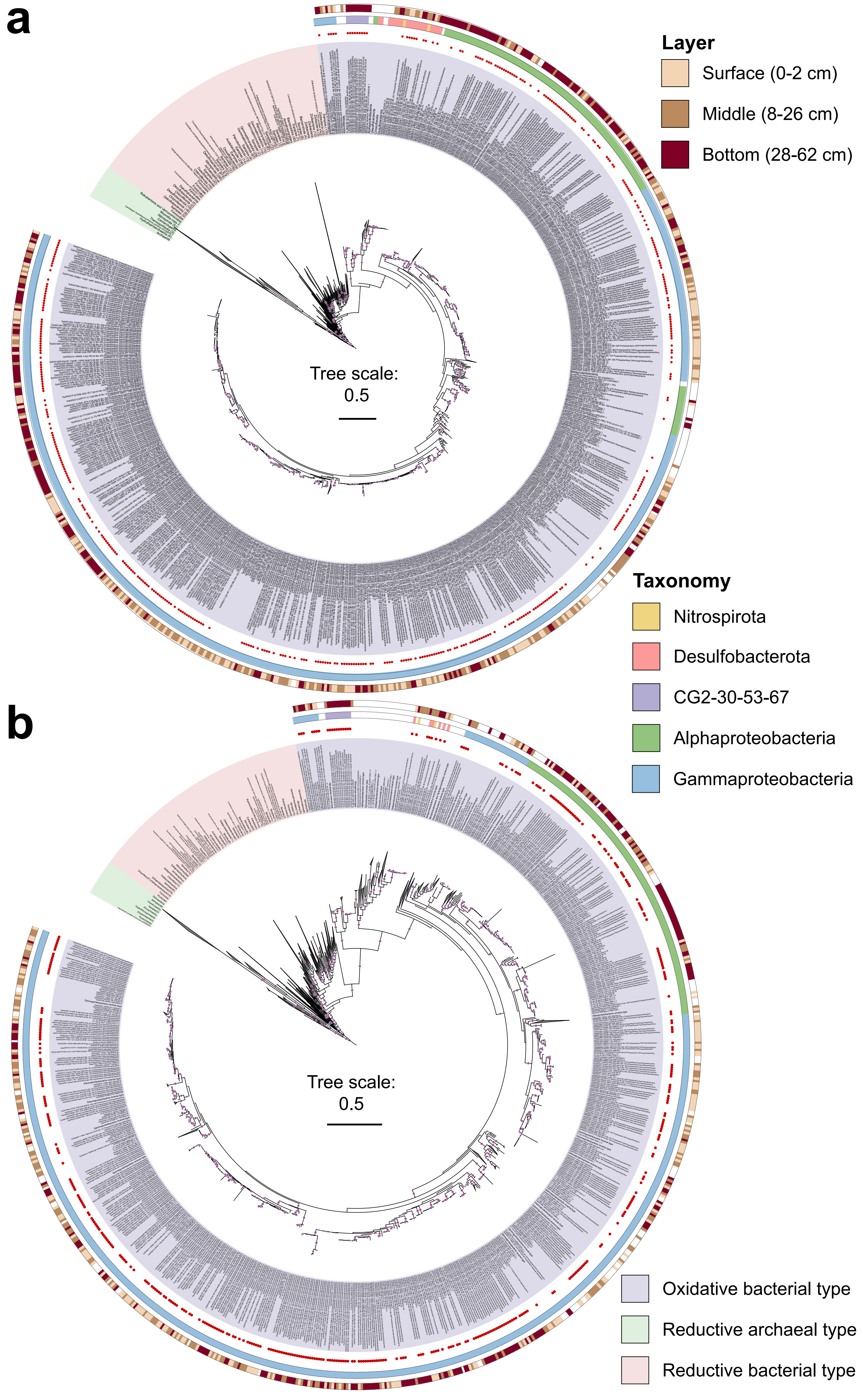
a
Layer
Surface (0-2 cm)
Middle (8-26 cm)
Bottom (28-62 cm)
Tree scale:
0.5
Taxonomy
Nitrospirota
Desulfobacterota
CG2-30-53-67
Alphaproteobacteria
Gammaproteobacteria
b
Tree scale:
0.5
Oxidative bacterial type
Reductive archaeal type
Reductive bacterial type

Supplementary Figure 6. Maximum likelihood phylogenetic tree of dissimilatory sulfite reductase (Dsr) protein sequences. (a) α subunit (DsrA) protein sequences of Dsr, including 2,621 sequences annotated in this study and 1,104 sequences downloaded from publicly available databases as reference. DsrA sequences from Aigarchaeota were rerooted as the outgroup. (b) β subunit (DsrB) sequences of Dsr, including 2,543 sequences annotated in this study and 1,514 sequences downloaded from publicly available databases as reference. DsrB sequences from Aigarchaeota were rerooted as the outgroup. Outer ring represents the sampling layer recovering the sequence. Middle ring represents the taxonomy. Inner dots represent sequences assigned into MAGs. Bootstrap values $\geq$80 are shown in circles. Sequences were aligned using MAFFT v7.475, trimmed using BMGE v1.12. The phylogenetic tree was constructed using IQ-TREE v1.6.12.

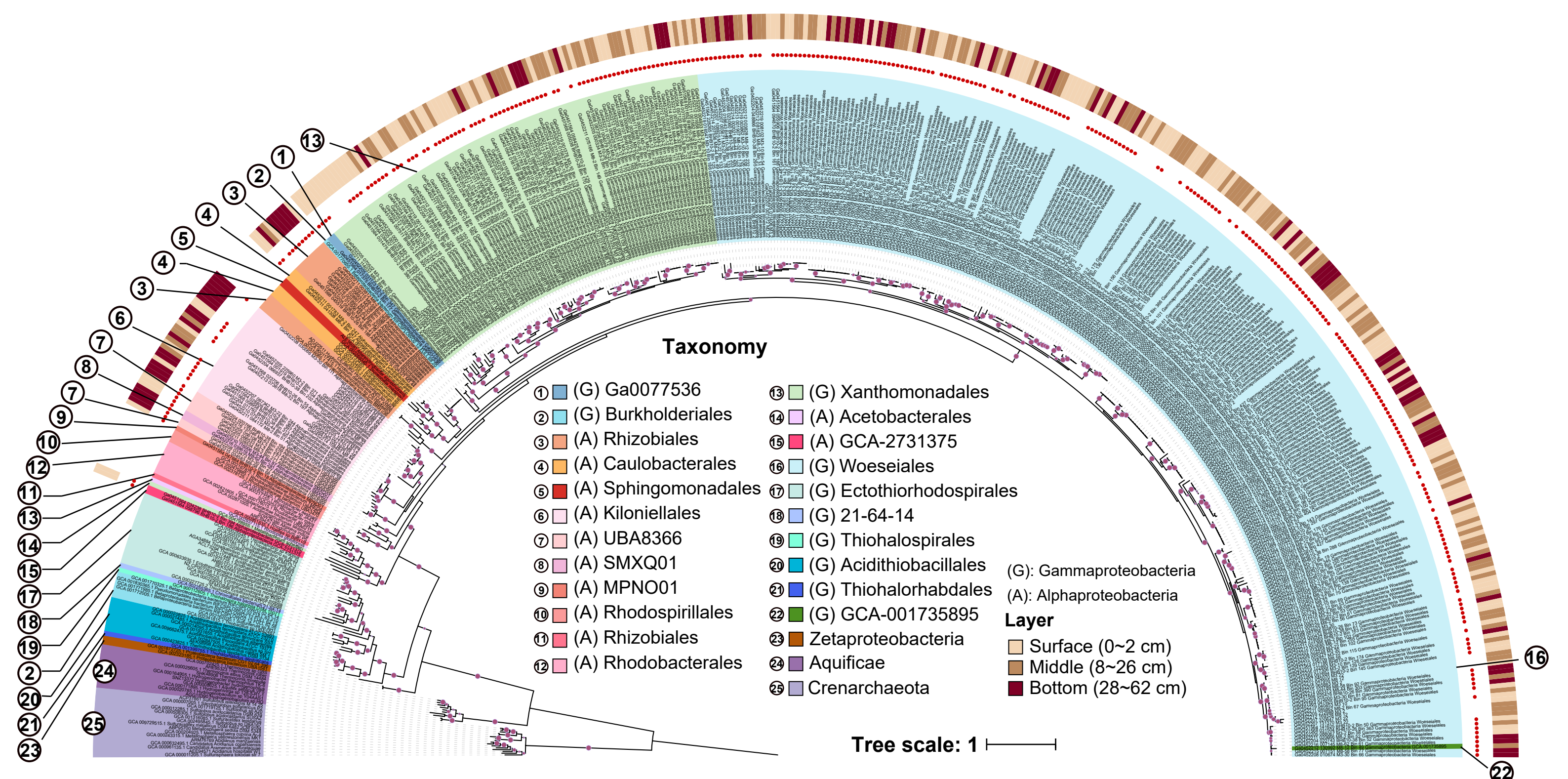
Taxonomy
① (G) Ga0077536
② (G) Burkholderiales
③ (A) Rhizobiales
④ (A) Caulobacterales
⑤ (A) Sphingomonadales
⑥ (A) Kiloniellales
⑦ (A) UBA8366
⑧ (A) SMXQ01
⑨ (A) MPNO01
⑩ (A) Rhodospirillales
⑪ (A) Rhizobiales
⑫ (A) Rhodobacterales
⑬ (G) Xanthomonadales
⑭ (A) Acetobacterales
⑮ (A) GCA-2731375
⑯ (G) Woeseiales
⑰ (G) Ectothiorhodospirales
⑱ (G) 21-64-14
⑲ (G) Thiohalospirales
⑳ (G) Acidithiobacillales
㉑ (G) Thiohalorhabdales
㉒ (G) GCA-001735895
㉓ Zetaproteobacteria
㉔ Aquificae
㉕ Crenarchaeota
(G): Gammaproteobacteria
(A): Alphaproteobacteria
Layer
Surface (0~2 cm)
Middle (8~26 cm)
Bottom (28~62 cm)
Tree scale: 1

Supplementary Figure 7. Maximum likelihood phylogenetic tree of Heterodisulfide reductase (Hdr)-like protein sequence clusters, including 406 clusters annotated in this study and 87 clusters downloaded from publicly available databases as reference. sHdr sequences from Crenarchaeota were rerooted as the outgroup. Outer ring represents the sampling layer recovering the sequence. Inner dots represent sequences assigned into MAGs. Bootstrap values $\geq$80 are shown in circles. Sequences were aligned using six different algorithms including MAFFT v7.450, ClustalW v2.1, DIALIGN v2.2.1, Muscle v3.8.31, Opal v2.1.3, and Kalign v3.3.1 with default settings. The alignment with highest consistency score for each subunit was selected using trimAl v1.2rev59. Phylogenetic tree was constructed using IQ-TREE v1.6.12.

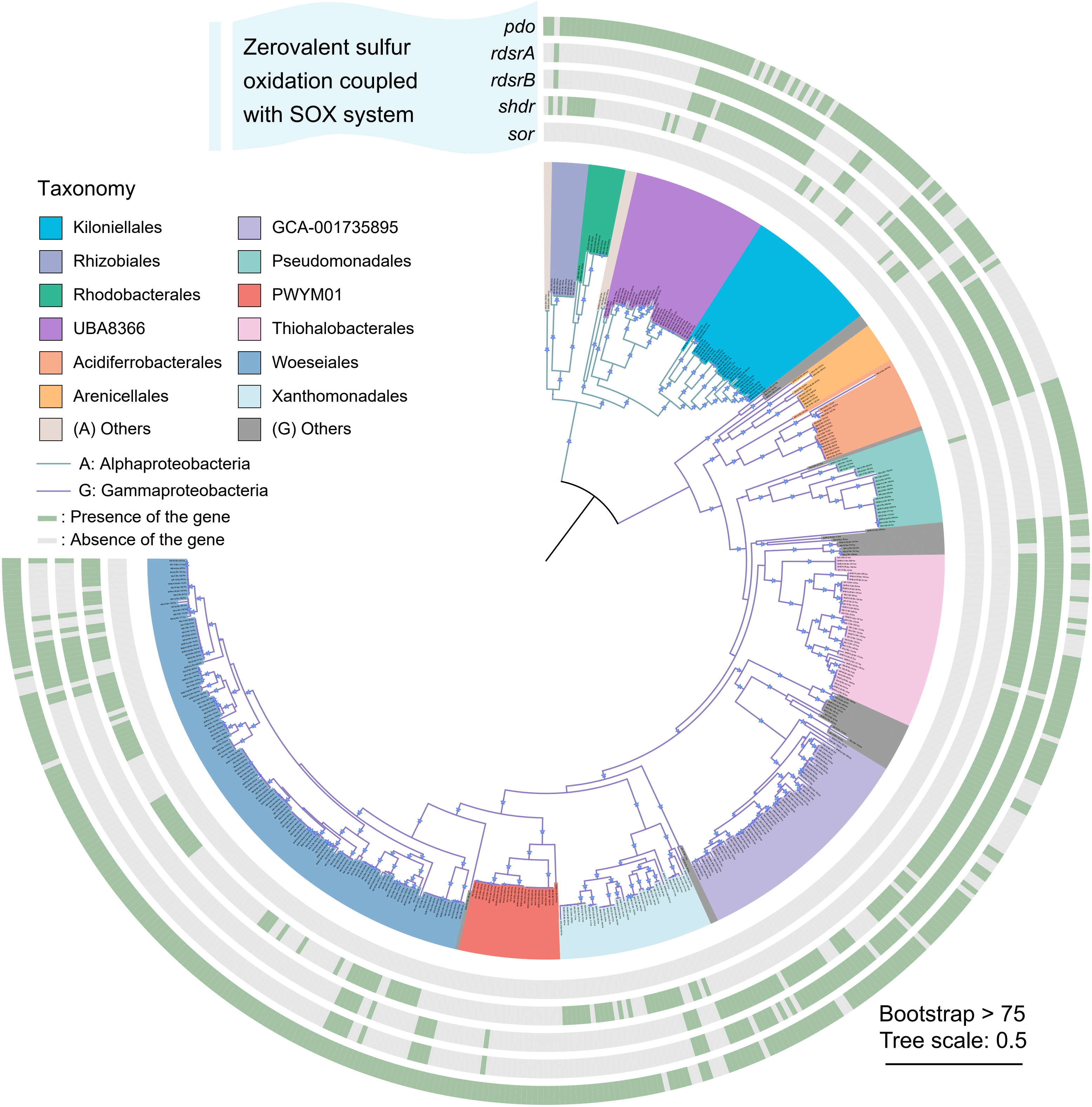

Zerovalent sulfur oxidation coupled with SOX system
pdo
rdsrA
rdsrB
shdr
sor
Taxonomy
Kiloniellales
Rhizobiales
Rhodobacterales
UBA8366
Acidiferrobacterales
Arenicellales
(A) Others
GCA-001735895
Pseudomonadales
PWYM01
Thiohalobacterales
Woeseiales
Xanthomonadales
(G) Others
A: Alphaproteobacteria
G: Gammaproteobacteria
: Presence of the gene
: Absence of the gene
Bootstrap > 75
Tree scale: 0.5

Supplementary Figure 8. Phylogeny of MAGs containing Sox system. A maximum likelihood phylogenetic tree of 460 MAGs based on 37 concatenated ribosomal protein encoding genes identified using PhyloSift. Sequences were aligned using MAFFT v7.471, trimmed using trimAl v1.4. The phylogenetic tree was constructed using IQ-TREE v2.1.2. Outer ring represents the taxonomy. Inner rings represent the presence of sHdr, PDO, rDsrA, rDsrB, or SOR protein sequence in MAGs from outer to the center.

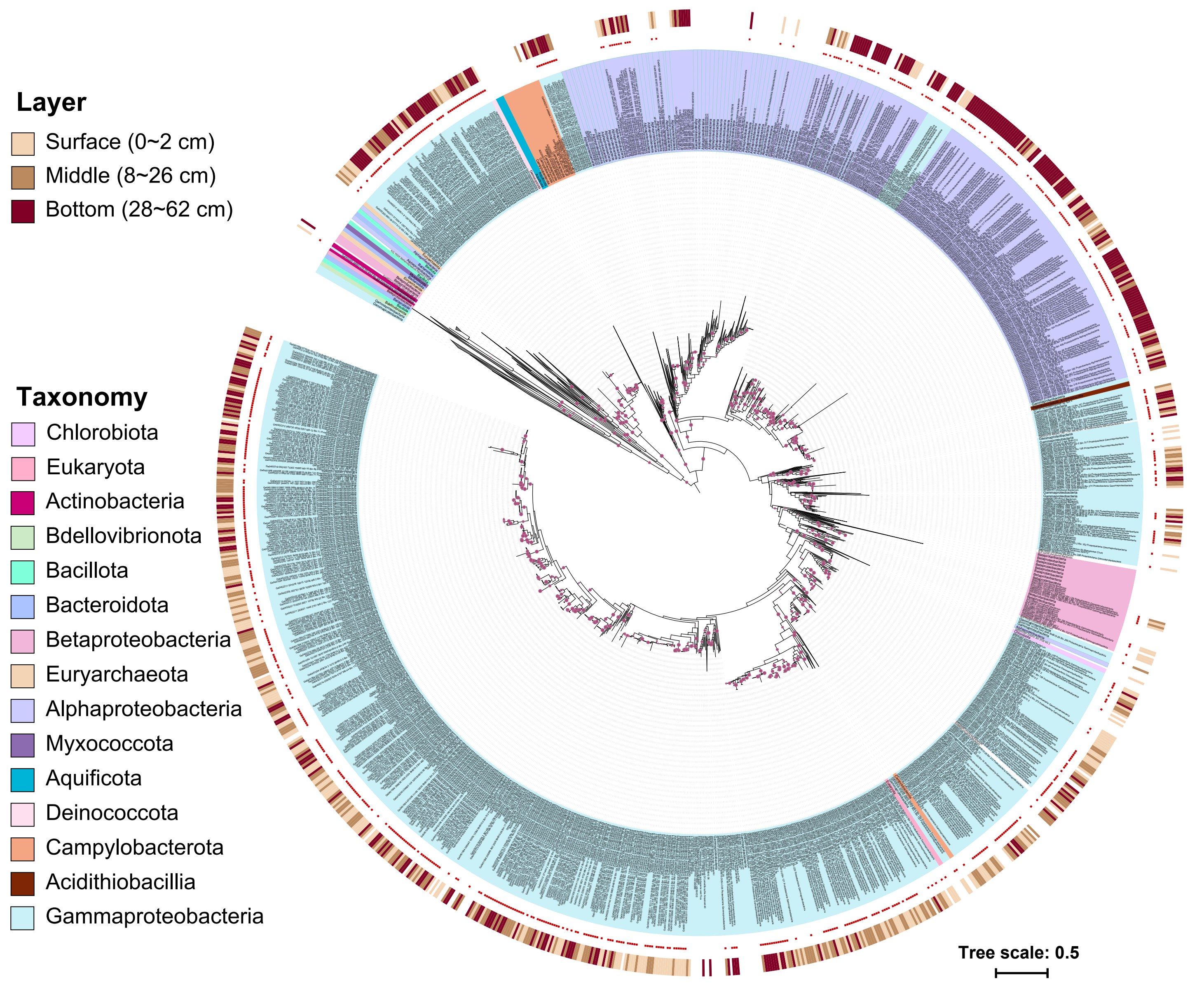
Layer
Surface (0~2 cm)
Middle (8~26 cm)
Bottom (28~62 cm)
Taxonomy
Chlorobiota
Eukaryota
Actinobacteria
Bdellovibrionota
Bacillota
Bacteroidota
Betaproteobacteria
Euryarchaeota
Alphaproteobacteria
Myxococcota
Aquificota
Deinococcota
Campylobacterota
Acidithiobacillia
Gammaproteobacteria
Tree scale: 0.5

Supplementary Figure 9. Maximum likelihood phylogenetic tree of catalytic subunits of thiosulfate oxidation multienzyme complex (SoxB) protein sequences, including 927 sequences annotated in this study and 1,752 sequences downloaded from publicly available databases as reference. SoxB sequences from order Enterobacterales were re-rooted as the outgroup. Outer ring represents the sampling layer recovering the sequence. Inner dots represent sequences assigned into MAGs. Bootstrap values ≥80 are shown in circles. Sequences were aligned using MAFFT v7.475, trimmed using BMGE v1.12. The phylogenetic tree was constructed using IQ-TREE v1.6.12.

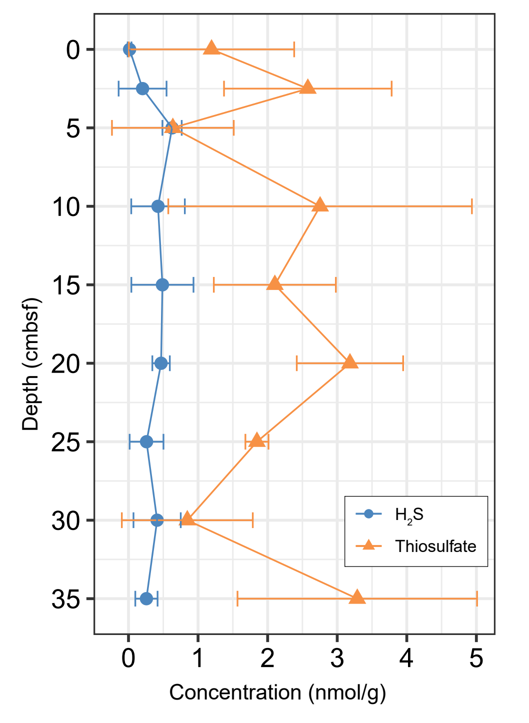

0
5
10
15
20
25
30
35
Depth (cmbsf)
0
1
2
3
4
5
Concentration (nmol/g)
H_2S
Thiosulfate

Supplementary Figure 10. Concentrations of sulfide and thiosulfate in different depths at station BHB10.

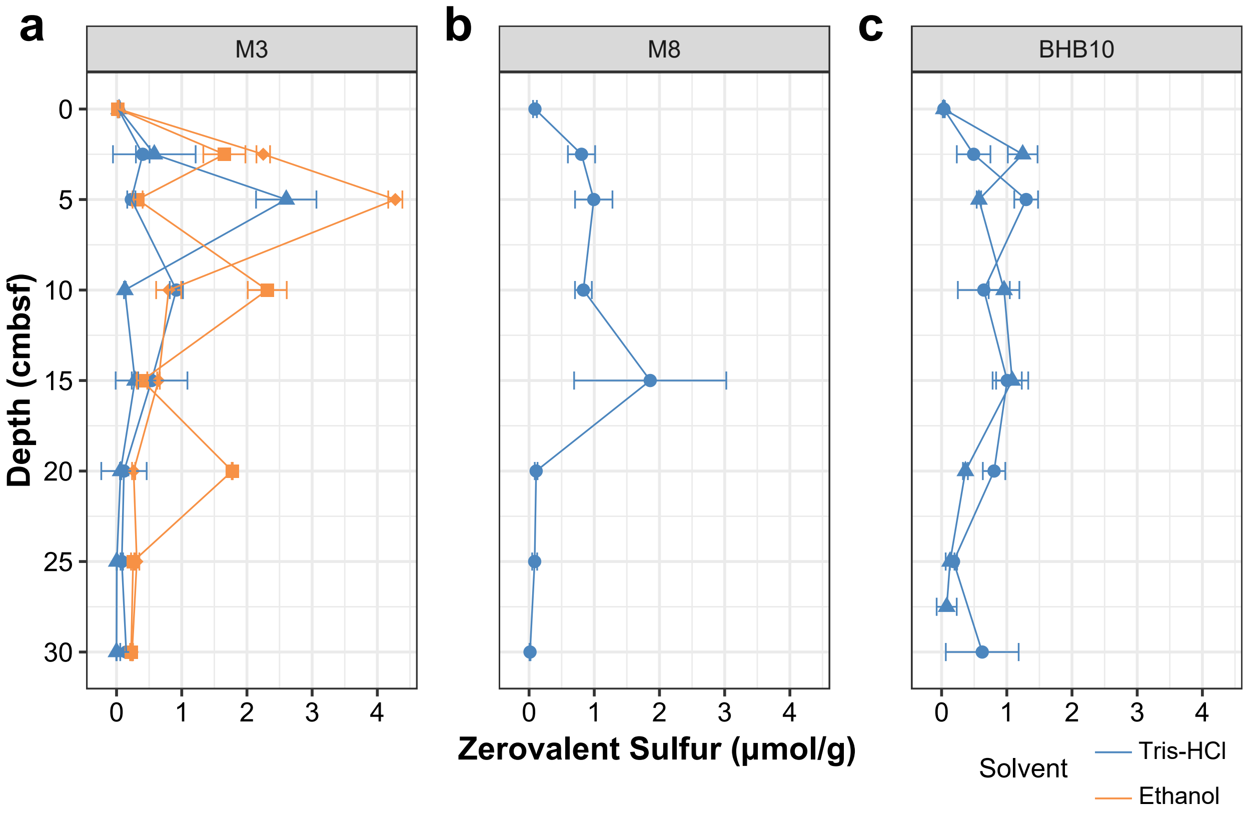

a
b
c
M3
M8
BHB10
Depth (cmbsf)
0
5
10
15
20
25
30
0
1
2
3
4
Zerovalent Sulfur (µmol/g)
Solvent
Tris-HCl
Ethanol

Supplementary Figure 11. Concentrations of zerovalent sulfur in different depths at three stations: M3 (a), M8 (b), and BHB10 (c). Sediment samples were stored with Tris-HCl buffer (orange) and ethanol (blue) for measurement of the concentration of zerovalent sulfur.

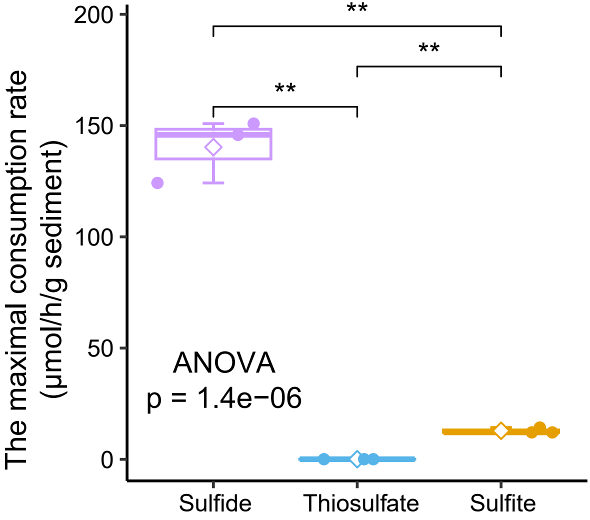

The maximal consumption rate
(μmol/h/g sediment)
200
150
100
50
0
**
**
**
ANOVA
p = 1.4e−06
Sulfide
Thiosulfate
Sulfite

Supplementary Figure 12. Statistical analysis of maximal rate of sulfur oxidation.

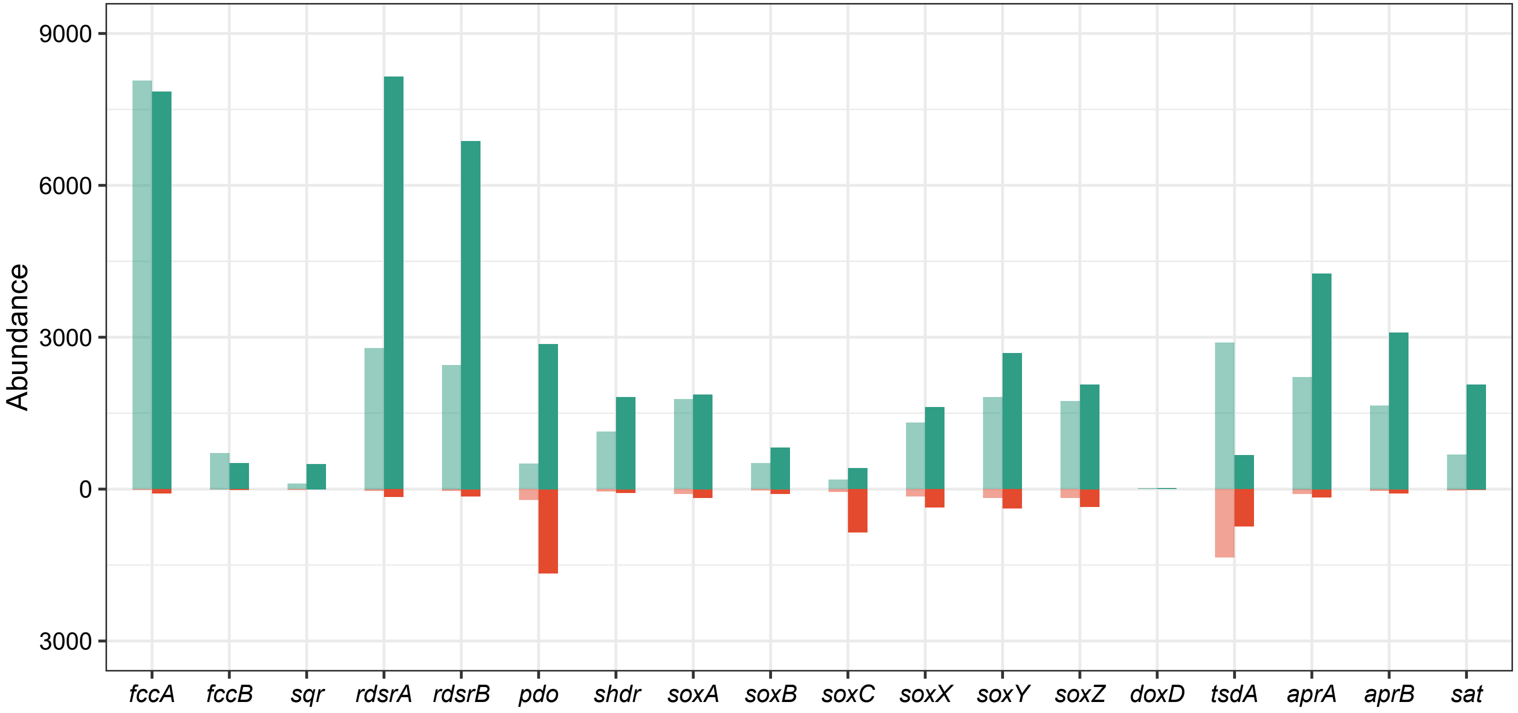

9000
6000
3000
0
3000
Abundance
fccA
fccB
sqr
rdsrA
rdsrB
pdo
shdr
soxA
soxB
soxC
soxX
soxY
soxZ
doxD
tsdA
aprA
aprB
sat

Supplementary Figure 13. Relative abundance of gene transcripts oxidizing sulfide, zerovalent sulfur, thiosulfate, and sulfite in Alphaproteobacteria and Gammaproteobacteria in control and experimental samples.

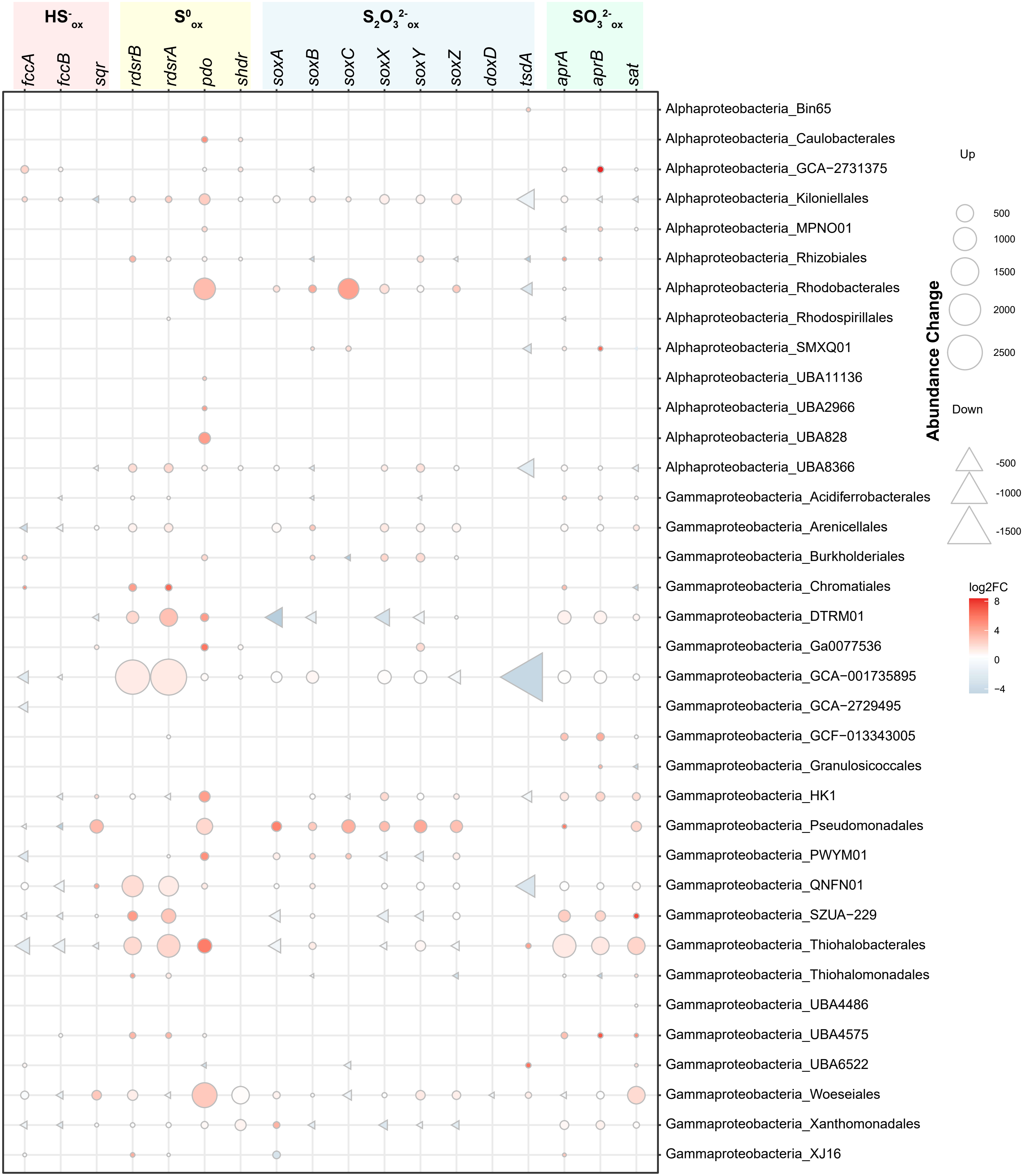

HS-ox
S0ox
S2O32-ox
SO32-ox
fccA
fccB
sqr
rdsrB
rdsrA
pdo
shdr
soxA
soxB
soxC
soxX
soxY
soxZ
doxD
tsdA
aprA
aprB
sat
Alphaproteobacteria_Bin65
Alphaproteobacteria_Caulobacterales
Alphaproteobacteria_GCA−2731375
Alphaproteobacteria_Kiloniellales
Alphaproteobacteria_MPNO01
Alphaproteobacteria_Rhizobiales
Alphaproteobacteria_Rhodobacterales
Alphaproteobacteria_Rhodospirillales
Alphaproteobacteria_SMXQ01
Alphaproteobacteria_UBA11136
Alphaproteobacteria_UBA2966
Alphaproteobacteria_UBA828
Alphaproteobacteria_UBA8366
Gammaproteobacteria_Acidiferrobacterales
Gammaproteobacteria_Arenicellales
Gammaproteobacteria_Burkholderiales
Gammaproteobacteria_Chromatiales
Gammaproteobacteria_DTRM01
Gammaproteobacteria_Ga0077536
Gammaproteobacteria_GCA−001735895
Gammaproteobacteria_GCA−2729495
Gammaproteobacteria_GCF−013343005
Gammaproteobacteria_Granulosicoccales
Gammaproteobacteria_HK1
Gammaproteobacteria_Pseudomonadales
Gammaproteobacteria_PWYM01
Gammaproteobacteria_QNFN01
Gammaproteobacteria_SZUA−229
Gammaproteobacteria_Thiohalobacterales
Gammaproteobacteria_Thiohalomonadales
Gammaproteobacteria_UBA4486
Gammaproteobacteria_UBA4575
Gammaproteobacteria_UBA6522
Gammaproteobacteria_Woeseiales
Gammaproteobacteria_Xanthomonadales
Gammaproteobacteria_XJ16
Abundance Change
Up
500
1000
1500
2000
2500
Down
-500
-1000
-1500
log2FC
8
4
0
−4

Supplementary Figure 14. Change of the relative abundance of gene transcripts oxidizing sulfide, zerovalent sulfur, thiosulfate, and sulfite at the order level within Alphaproteobacteria and Gammaproteobacteria after the addition of NaHS.

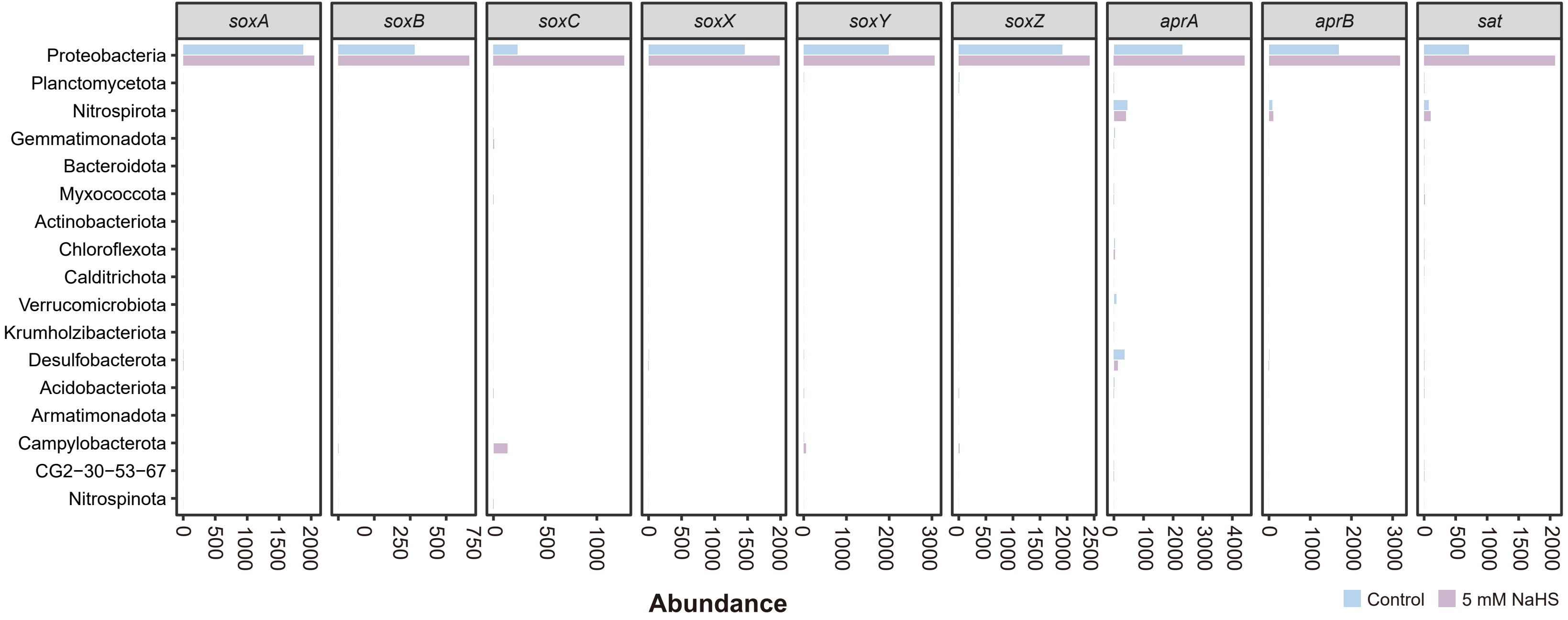

soxA
soxB
soxC
soxX
soxY
soxZ
aprA
aprB
sat
Proteobacteria
Planctomycetota
Nitrospirota
Gemmatimonadota
Bacteroidota
Myxococcota
Actinobacteriota
Chloroflexota
Calditrichota
Verrucomicrobiota
Krumholzibacteriota
Desulfobacterota
Acidobacteriota
Armatimonadota
Campylobacterota
CG2−30−53−67
Nitrospinota
Abundance
Control
5 mM NaHS

Supplementary Figure 15. Relative abundance of *sox* and *arpAB* transcripts at the phylum level in control and experimental samples.

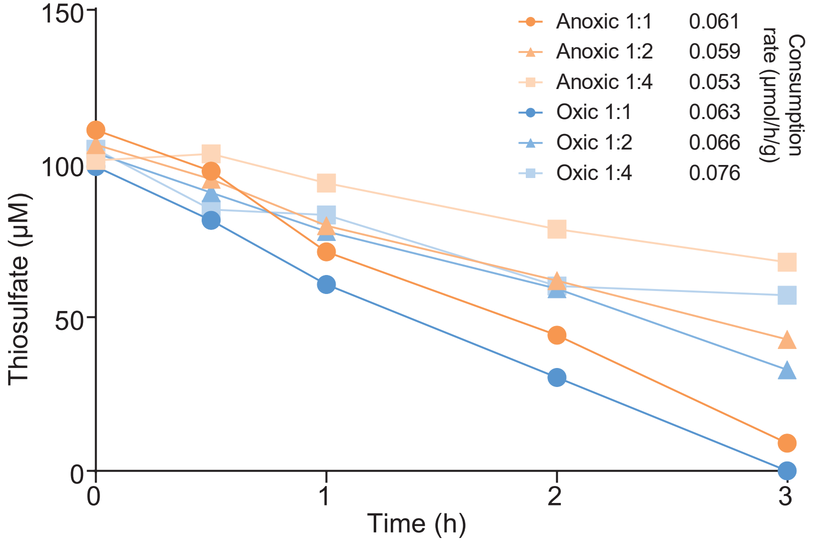

Thiosulfate (µM)
150
100
50
0
0
1
2
3
Time (h)
Anoxic 1:1 0.061
Anoxic 1:2 0.059
Anoxic 1:4 0.053
Oxic 1:1 0.063
Oxic 1:2 0.066
Oxic 1:4 0.076
Consumption rate (µmol/h/g)

Supplementary Figure 16. Time course of the concentrations of thiosulfate during the incubation of diluted samples at 25 ℃ on a shaker (200 rpm) with the addition of thiosulfate solution to the final concentration of 100 μmol/L under oxic and anoxic conditions. 5g sediments were diluted with 5 ml (1:1), 10 ml (1:2), and 20 ml (1:4) sterilized seawater, respectively.
